# Supplementary material for: An isolable, chelating bis[cyclic (alkyl)(amino)carbene] stabilizes a strongly bent, dicoordinate Ni(0) complex
Source: Nat Commun. 2024 Apr 23;15:3417. doi: 10.1038/s41467-024-47036-7 (PMC11039645; doi:10.1038/s41467-024-47036-7)
Supplement: Supplementary file 1 — Supplementary Information [file 41467_2024_47036_MOESM1_ESM.pdf]

An isolable, chelating bis[cyclic (alkyl)(amino)carbene] stabilizes a strongly bent, dicoordinate Ni(o) complex

Braulio M. Puerta Lombardi, Morgan R. Faas, Daniel West, Roope A. Suvinen, Heikki M. Tuononen,* Roland Roesler*

Supplementary Information

Table of Contents

Synthetic Details 3

General Considerations 3

Synthesis of C_2-symmetric bis(iminium) salt 2 (racemic) 4

Synthesis of C_2-symmetric bis(CAAC) 3 (racemic) 4

Synthesis of iron(II) complex 4 5

Synthesis of cobalt(II) complex 5 6

Synthesis of zinc(II) complex 6 6

Synthesis of nickel(II) complex 7 7

Synthesis of nickel(0) complex 8 7

Synthesis of nickel(I) complex 9 8

NMR Spectra 9

Supplementary Fig. 1. ^{1}H NMR spectrum of **2** 9

Supplementary Fig. 2. ^{13}C DEPTQ NMR spectrum of **2** 10

Supplementary Fig. 3. ^{1}H-^{13}C HSQC NMR spectrum of **2** 11

Supplementary Fig. 4. ^{1}H-^{13}C HMBC NMR spectrum of **2** 12

Supplementary Fig. 6. ^{13}C DEPTQ NMR spectrum of **3** 14

Supplementary Fig. 7. ^{1}H-^{13}C HSQC spectrum of **3** 15

Supplementary Fig. 8. ^{1}H-^{13}C HMBC spectrum of **3** 16

Supplementary Fig. 9. ^{1}H NMR spectrum of **6** 17

Supplementary Fig. 10. ^{13}C NMR spectrum of **6** 18

Supplementary Fig. 11. ^{1}H-^{13}C HSQC NMR spectrum of **6** 19

Supplementary Fig. 12. ^1H-^{13}C HMBC NMR spectrum of **6** ... 20
Supplementary Fig. 13. ^1H NMR spectrum of **8** ... 21
Supplementary Fig. 14. Low temperature ^1H NMR spectrum of **8** ... 22
Supplementary Fig. 15. Low temperature ^{13}C DEPTQ NMR spectrum of **8** ... 23
Supplementary Fig. 16. Low temperature ^1H-^{13}C HSQC spectrum of **8** ... 24
Supplementary Fig. 17. Low temperature ^1H-^{13}C HMBC spectrum of **8** ... 25
Crystallographic Details ... 26
Supplementary Table 1. Summary of Crystallographic Data for Compounds **3** and **4–9** ... 26
Supplementary Table 1. Continued ... 27
Supplementary Fig. 18. Solid-state structure of **3** ... 28
Supplementary Fig. 19. Solid-state structure of **4** ... 28
Supplementary Fig. 20. Solid-state structure of **5** ... 29
Supplementary Fig. 21. Solid-state structure of **6** ... 29
Supplementary Fig. 22. Solid-state structure of **7** ... 30
Supplementary Fig. 23. Solid-state structure of **8** ... 30
Supplementary Fig. 24. Solid-state structure of **9** ... 31
Supplementary Fig. 25. Solid-state structures of **4–7** viewed along the molecular (non-crystallographic) C_2-symmetry axis ... 32
Supplementary Fig. 26. Space-filling representations of solid-state structures of **7** (top), **8** (middle), and **9** (bottom) ... 33
Computational Details ... 34
General Considerations ... 34
Energy Decomposition Analyses ... 34
Homologues of Ligand 8 ... 35
Supplementary Table 2. Summary of Results from Energy Decomposition Analysis of Metal–Ligand Bonding ... 36
Supplementary Fig. 27. Simplified Walsh diagram for bending an L–M–L complex (L = π-acceptor ligand, M = d^{10}-metal) ... 36
Supplementary Fig. 28. Canonical frontier Kohn-Sham orbitals ... 37
Supplementary References ... 38

Synthetic Details

General Considerations

Unless otherwise stated, synthesis and handling of all compounds was performed under strict exclusion of air and moisture in an argon atmosphere, using a double-manifold vacuum line and an MBRAUN glove box operating with argon. Pentane was dried using an MBRAUN solvent purification system and stored in a 500 mL air-tight glass vessel containing sodium. Benzene, toluene, and tetrahydrofuran (THF) were dried over potassium, distilled for storage into 500 mL air-tight vessels containing sodium/benzophenone ketyl, and vacuum-transferred into the reaction vessel. Acetonitrile and dichloromethane were dried over calcium hydride and stored in 500 mL air-tight vessels over 4 Å molecular sieves. 1,3-diiodopropane (Oakwood Chemicals), $FeCl_2$, $CoCl_2$, $ZnCl_2$ (Alfa-Aesar), $NiBr_2$(dme) (Millipore-Sigma), and all other reagents (Millipore-Sigma, Oakwood Chemicals) were used as received. The enamine precursor was synthesized according to a literature procedure,[1] and was passed through a silica plug in hexanes before use. Sigman's bromide dimer was synthesized by following a reported procedure.[2]

Nuclear magnetic resonance (NMR) spectra were acquired on Bruker Avance and Avance III 400 MHz spectrometers at 298 K, unless otherwise noted. ^{1}H and ^{13}C NMR chemical shifts were referenced to residual solvent peaks and naturally abundant ^{13}C resonances for all deuterated solvents: $CHCl_3$ (7.26 ppm, ^{1}H) and $CHCl_3$-d_1 (77.16 ppm, ^{13}C); CH_2Cl_2-d_1 (5.32 ppm, ^{1}H) and CH_2Cl_2-d_2 (54.00 ppm, ^{13}C); THF-d_7 (3.58 ppm, ^{1}H) and THF-d_8 (67.21 ppm, ^{13}C); benzene-d_5 (7.16 ppm, ^{1}H) and benzene-d_6 (128.06 ppm, ^{13}C).[3]

X-ray crystallographic data were collected on a Bruker SMART APEX II CCD diffractometer using suitable single crystals coated in Paratone 8277 oil (Exxon) and mounted on glass-fiber loops. Measurements were processed with the Apex III software suite. Structures were solved using the SHELXT[4] structure solution program with intrinsic phasing and refined using the SHELXL[5] refinement package with least squares minimization, all under the Olex2 platform.[6] Full crystallographic details can be found in each independently uploaded crystallographic information file (cif).

All elemental analyses were obtained on a Perkin-Elmer Model 2400 Series II analyzer. High resolution electrospray mass spectra (HRESI-MS) were obtained with a Kratos MS-80 spectrometer using samples prepared in the glovebox and transferred in a gas-tight syringe.

Synthesis of C_2-symmetric bis(iminium) salt 2 (racemic)

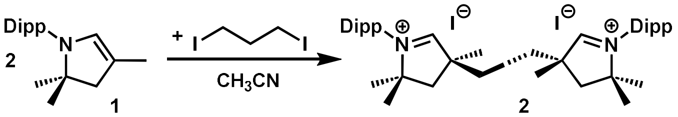

In an argon glovebox, a 150 mL air-tight flask containing a stir bar was charged with 1,3-diiodopropane (2.00 g, 6.76 mmol), the enamine precursor **1** (5.50 g, 20.3 mmol), and anhydrous acetonitrile (15 mL). The flask was sealed and heated outside the glovebox at 100 °C for 72 h, leading to the formation of an off-white precipitate. The flask was opened to air, the mixture was filtered, and the solid was washed with acetonitrile (3 × 10 mL) followed by diethyl ether (2 × 10 mL) and dried in vacuo. Diiodide salt **2** was obtained as a white powder (1.50 g, 1.79 mmol, 27 % yield). Anal. Calcd. for $C_{41}H_{64}N_2I_2$: C 58.71; H 7.69; N 3.34. Found: C 58.82; H 7.61; N 3.29. **^{1}H NMR** (CD_2Cl_2, 25 °C, 400 MHz): δ 1.24 (d, $^3J_{HH}$ = 6.7 Hz, 3H, CH(C***H***$_3$)$_2$), 1.28 (d, $^3J_{HH}$ = 6.7 Hz, 3H, CH(C***H***$_3$)$_2$), 1.35 (d, $^3J_{HH}$ = 6.7 Hz, 3H, CH(C***H***$_3$)$_2$), 1.40 (d, $^3J_{HH}$ = 6.7 Hz, 3H, CH(C***H***$_3$)$_2$), 1.53 (s, 3H, C(C***H***$_3$)$_2$), 1.63 (s, 3H, C(C***H***$_3$)$_2$), 1.76 (s, 3H, C***H***$_3$), 2.07 (m, 1H, CCH$_2$C***H***$_2$), 2.23 (m, 2H, CC***H***$_2$CH$_2$), 2.41 (d, $^2J_{HH}$ = 13.9 Hz, 1H, C***H***$_2$), 2.62 (sept, $^3J_{HH}$ = 6.8 Hz, 1H, C***H***(CH$_3$)$_2$), 2.75 (sept, $^3J_{HH}$ = 6.8 Hz, 1H, C***H***(CH$_3$)$_2$), 2.83 (d, $^2J_{HH}$ = 13.9 Hz, 1H, C***H***$_2$), 7.38 (m, 2H, *m*-C$_6$***H***$_3$), 7.56 (vt, J = 7.8 Hz, 1H, *p*-C$_6$***H***$_3$), 10.51 (s, 1H, C***H***=N). **^{13}C NMR** (CD_2Cl_2, 25 °C, 101 MHz): δ 21.9 (s, CCH$_2$***C***H$_2$), 22.2 (s, CH(***C***H$_3$)$_2$), 22.4 (s, CH(***C***H$_3$)$_2$), 24.7 (s, ***C***H$_3$), 26.9 (s, CH(***C***H$_3$)$_2$), 27. 1 (s, CH(***C***H$_3$)$_2$), 28.7 (s, C(***C***H$_3$)$_2$), 28.8 (s, C(***C***H$_3$)$_2$), 30.1 (s, ***C***H(CH$_3$)$_2$), 30.2 (s, ***C***H(CH$_3$)$_2$), 39.8 (s, C***C***H$_2$CH$_2$), 48. 2 (s, ***C***H$_2$), 52.4 (s, ***C***(CH$_3$)CH$_2$), 83.8 (s, ***C***(CH$_3$)$_2$), 125.6 (s, *m*-***C***$_6$H$_3$), 125.8 (s, *m*-***C***$_6$H$_3$), 129.0 (s, *i*-***C***$_6$H$_3$), 132.2 (s, *p*-***C***$_6$H$_3$), 144.6 (s, *o*-***C***$_6$H$_3$), 144.8 (s, *o*-***C***$_6$H$_3$), 191.7 (s, ***C***H=N).

The salt **2** is a fluffy, white powder that did not appear to be particularly hygroscopic or air-sensitive by ^{1}H NMR but was stored in an argon glovebox out of an abundance of caution. Compound **2** is soluble in dichloromethane, chloroform, and 1,2-difluorobenzene, sparingly soluble in THF, 1,4-dioxane, and acetonitrile, and insoluble in hydrocarbon solvents and diethyl ether.

Synthesis of C_2-symmetric bis(CAAC) 3 (racemic)

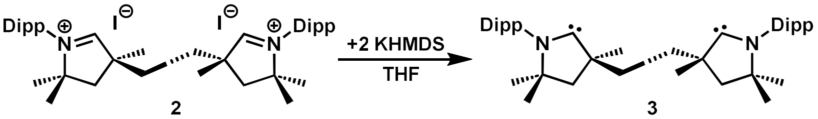

In an argon glovebox, a 50 mL round bottomed flask containing a stir bar was charged with **2** (2.00 g, 2.39 mmol) and KHMDS (0.998 g, 5.00 mmol). The flask was attached to a swivel frit and 30 mL of THF was vacuum transferred to the mixture, which was then stirred under argon for three hours. The solvent was subsequently removed in vacuo and the residue was extracted with benzene (2 × 20 mL) and filtered. After removing benzene in vacuo, the crude product was washed with cold pentane and dried under high vacuum to yield **3** (1.11 g, 1.89 mmol, 79.1 %) as a white powder. X-ray quality crystals were obtained *via* slow evaporation of a pentane solution. Anal.

Calcd. for $C_{41}H_{62}N_2$: C 84.47; H 10.72; N 4.81. Found: C 84.71; H 10.88; N 4.71. **^{1}H NMR** (C_6D_6, 25 °C, 400 MHz): δ 1.14 (s, 3H, C(C***H***$_3$)$_2$), 1.15 (s, 3H, C(C***H***$_3$)$_2$), 1.23 (d, $^3J_{HH}$ = 6.8 Hz, 3H, CH(C***H***$_3$)$_2$), 1.25 (d, $^3J_{HH}$ = 6.8 Hz, 3H, CH(C***H***$_3$)$_2$), 1.25 (d, $^3J_{HH}$ = 6.8 Hz, 3H, CH(C***H***$_3$)$_2$), 1.29 (d, $^3J_{HH}$ = 6.8 Hz, 3H, CH(C***H***$_3$)$_2$), 1.43 (s, 3H, C***H***$_3$), 1.51 (d, $^2J_{HH}$ = 12.9 Hz, 1H, C***H***$_2$), 1.79 (d, $^2J_{HH}$ = 12.9 Hz, 1H, C***H***$_2$), 1.85 (m, 1H, CCH$_2$C***H***$_2$), 2.00 (m, 2H, CC***H***$_2$CH$_2$), 3.19 (sept, $^3J_{HH}$ = 6.8 Hz, 1H, C***H***(CH$_3$)$_2$), 3.23 (sept, $^3J_{HH}$ = 6.8 Hz, 1H, C***H***(CH$_3$)$_2$), 7.15 (m, 2H, *m*-C$_6$***H***$_3$), 7.22 (vt, J = 7.6 Hz, 1H, *p*-C$_6$***H***$_3$). **^{13}C NMR** (C_6D_6, 25 °C, 101 MHz): δ 21.8 (s, CCH$_2$***C***H$_2$), 21.9, 22.0, 25.7, 26.3, 26.4, 29.2 (s, ***C***H(CH$_3$)$_2$), 29.3 (s, ***C***H(CH$_3$)$_2$), 29.4 (s, C(***C***H$_3$)$_2$), 29.7 (s, C(***C***H$_3$)$_2$), 42.9 (s, C***C***H$_2$CH$_2$), 48.0 (s, ***C***H$_2$), 62.5 (s, ***C***(CH$_3$)CH$_2$), 82.2 (s, ***C***(CH$_3$)$_2$), 123.8 (s, *m*-***C***$_6$H$_3$), 124.0 (s, *m*-***C***$_6$H$_3$), 128.1 (s, *p*-***C***$_6$H$_3$), 138.1 (s, *i*-***C***$_6$H$_3$), 146.1 (s, *o*-***C***$_6$H$_3$), 146.3 (s, *o*-***C***$_6$H$_3$), 314.7 (s, ***C***$_{carbene}$).

Biscarbene **3** is a highly air and moisture sensitive, fluffy white solid that was handled in an argon glovebox. The stability of **3** was similar to that of other CAACs. No signs of decomposition were observed after storing the solid in a scintillation vial in the glovebox freezer (−40 °C) for 1.5 months. Decomposition becomes noticeable by ^{1}H NMR when solutions of the compound in benzene are allowed to sit at room temperature for more than 24 hours. The nature of the decomposition products has not yet been elucidated, but NMR data suggests intramolecular C–H activation at the diisopropylphenyl-methyne carbon.

Compound **3** is very soluble in THF, 1,4-dioxane, toluene, and benzene, and sparingly soluble in pentane. Deprotonation was quantitative by ^{1}H NMR, however, since **2** is not very soluble in THF, preparative-scale deprotonation is slow, requiring at least three hours. Due to the high solubility of **3** in benzene, removal of this solvent in vacuum may yield an oil. Crystallization of the compound can be initiated by triturating with pentane around four times (pentane added, mixture sonicated, and pentane evaporated without filtering) to help remove all volatiles. The off-white solid can be washed with cold pentane and filtered with minimal product loss.

Synthesis of iron(II) complex 4

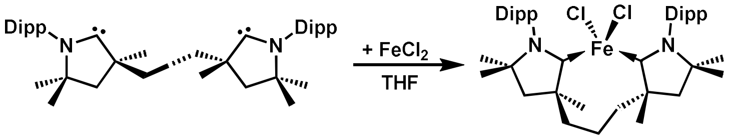

3 4

In an argon glovebox, a 25 mL round bottom flask containing a stir bar was charged with **3** (0.2 g, 0.341 mmol) and anhydrous $FeCl_2$ (0.043 g, 0.339 mmol). The flask was attached to a swivel frit apparatus and 10 mL of THF was vacuum transferred on top of the solids. The mixture was stirred under argon overnight and subsequently filtered. The solid was dried under high vacuum to yield **4** (0.151 g, 0.213 mmol, 63 %) as a yellow powder. X-ray quality crystals were obtained by allowing the THF reaction filtrate to stand at room temperature overnight. Magnetic susceptibility μ_{eff} was determined to be 4.78 (4 unpaired electrons) at 298 K using Evans method. The chemical shift of Si(SiMe$_3$)$_4$ and a 0.013 M solution of **4** in CD_2Cl_2 were used. Anal. Calcd. for $C_{41}H_{62}N_2Cl_2Fe$: C 69.39; H 8.81; N 3.95. Found: C 69.31; H 9.19; N 3.86. HRMS (ESI) m/z: $[M+H]^+$ Calcd. for $C_{41}H_{62}N_2Cl_2Fe$ 709.3712; Found: 709.3715.

Synthesis of cobalt(II) complex 5

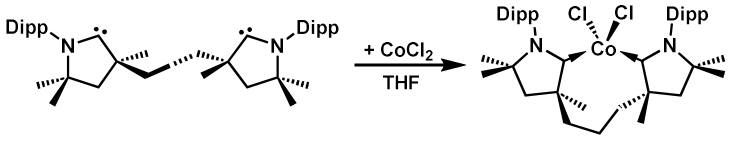

3 5

In an argon glovebox, a 25 mL round bottomed flask containing a stir bar was charged with dicarbene **3** (0.2 g, 0.341 mmol) and anhydrous $CoCl_2$ (0.044 g, 0.339 mmol). The flask was attached to a swivel frit apparatus and 10 mL of THF was vacuum transferred on top of the solids. The mixture was stirred under argon overnight and subsequently filtered. The solid was dried under high vacuum to yield **5** (0.147 g, 0.206 mmol, 61 %) as a blue powder. X-ray quality crystals were obtained by allowing the THF reaction filtrate to stand at room temperature overnight. Magnetic susceptibility μ_{eff} was determined to be 4.27 (3 unpaired electrons) at 298 K using Evans method. The chemical shift of $Si(SiMe_3)_4$ and a 0.02 M solution of **5** in CD_2Cl_2 were used. Anal. Calcd. for $C_{41}H_{62}N_2Cl_2Co$: C 69.09; H 8.77; N 3.93. Found: C 68.89; H 9.01; N 3.82. HRMS (ESI) m/z: $[M+H]^+$ Calcd. for $C_{41}H_{62}N_2Cl_2Co$ 712.3695; Found: 712.3700.

Synthesis of zinc(II) complex 6

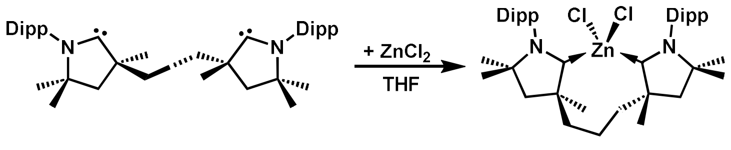

3 6

In an argon glovebox, a 25 mL round bottom flask containing a stir bar was charged with dicarbene **3** (0.205 g, 0.349 mmol) and anhydrous $ZnCl_2$ (0.048 g, 0.352 mmol). The flask was attached to a swivel frit apparatus and 10 mL of THF was vacuum transferred on top of the solids. The mixture was stirred under argon overnight and subsequently filtered. The solid was dried under high vacuum to yield **6** (0.200 g, 0.278 mmol, 80 %) as a white powder. X-ray quality crystals were obtained by allowing the THF reaction filtrate to stand at room temperature overnight. **Anal.** Calcd. for $C_{41}H_{62}N_2Cl_2Zn$: C 68.47; H 8.69; N 3.89. Found: C 68.35; H 9.13; N 3.79. **^{1}H NMR** (CD_2Cl_2, 25 °C, 400 MHz): δ 1.20 (d, $^3J_{HH}$ = 6.6 Hz, 3H, CH(C***H***$_3$)$_2$), 1.30 (d, $^3J_{HH}$ = 6.4 Hz, 3H, CH(C***H***$_3$)$_2$), 1.32 (s, 3H, C(C***H***$_3$)$_2$), 1.38 (d, $^3J_{HH}$ = 6.4 Hz, 3H, CH(C***H***$_3$)$_2$), 1.38 (s, 3H, C(C***H***$_3$)$_2$), 1.51 (d, $^3J_{HH}$ = 6.6 Hz, 3H, CH(C***H***$_3$)$_2$), 1.61 (m, 1H, CC***H***$_2$CH$_2$), 1.65 (s, 3H, C***H***$_3$), 1.71 (d, $^2J_{HH}$ = 12.9 Hz, 1H, C***H***$_2$), 1.81 (m, 1H, CCH$_2$C***H***$_2$), 2.09 (m, 1H, CC***H***$_2$CH$_2$), 2.16 (d, $^2J_{HH}$ = 12.9 Hz, 1H, C***H***$_2$), 2.74 (sept, $^3J_{HH}$ = 6.4 Hz, 1H, C***H***(CH$_3$)$_2$), 2.82 (sept, $^3J_{HH}$ = 6.6 Hz, 1H, C***H***(CH$_3$)$_2$), 7.24 (m, 2H, *m*-C$_6$***H***$_3$), 7.39 (vt, J = 7.73 Hz, 1H, *p*-C$_6$***H***$_3$). **^{13}C NMR** (CD_2Cl_2, 25 °C, 101 MHz): δ 24.6 (s, CH(***C***H$_3$)$_2$), 24.7 (s, CH(***C***H$_3$)$_2$), 26.0 (s, CCH$_2$***C***H$_2$), 28.0 (s, CH(***C***H$_3$)$_2$), 28.3 (s, ***C***H(CH$_3$)$_2$), 28.8 (s, CH(***C***H$_3$)$_2$), 29.4 (s, C(***C***H$_3$)$_2$), 29.8 (s,***C***H$_3$), 30.1 (s, ***C***H(CH$_3$)$_2$), 30.9 (s, C(***C***H$_3$)$_2$), 38.4 (s, C***C***H$_2$CH$_2$), 43.6 (s, ***C***H$_2$), 60.7 (s, ***C***(CH$_3$)CH$_2$), 83.1 (s, ***C***(CH$_3$)$_2$), 124.5 (s, *m*-***C***$_6$H$_3$), 125.4 (s, *m*-***C***$_6$H$_3$), 129.1 (s, *p*-***C***$_6$H$_3$), 134.1 (s, *i*-***C***$_6$H$_3$), 145.5 (s, *o*-***C***$_6$H$_3$), 146.9 (s, *o*-***C***$_6$H$_3$), 255.4 (s, ***C***$_{carbene}$).

Synthesis of nickel(II) complex 7

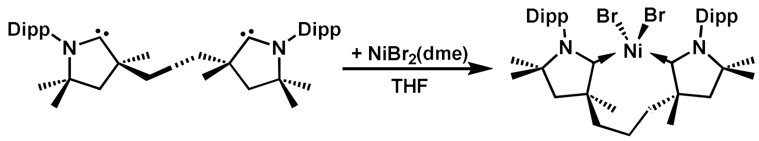

3 **7**

In an argon glovebox, a 25 mL round bottomed flask containing a stir bar was charged with dicarbene **3** (0.5 g, 0.852 mmol) and $NiBr_2$(dme) (0.263 g, 0.852 mmol). The flask was attached to a swivel frit apparatus and 15 mL of THF was vacuum transferred on top of the solids. The mixture was stirred under argon overnight and subsequently filtered. The solid was dried under high vacuum to yield **7** (0.483 g, 0.603 mmol, 71 %) as a light blue powder. X-ray quality crystals were obtained *via* slow-diffusion of pentane into a solution of **7** in 1,2-difluorobenzene. Magnetic susceptibility μ_{eff} was determined to be 3.04 (2 unpaired electrons) at 298 K using Evans method. The chemical shift of $Si(SiMe_3)_4$ and a 0.012 M solution of **7** in CD_2Cl_2 were used. Anal. Calcd. for $C_{41}H_{62}N_2Br_2Ni$: C 61.44; H 7.80; N 3.50. Found: C 61.44; H 8.07; N 3.41. HRMS (ESI) m/z: $[M+H]^+$ Calcd. for $C_{41}H_{62}N_2Br_2Ni$ 801.2686; Found: 801.2659.

Synthesis of nickel(0) complex 8

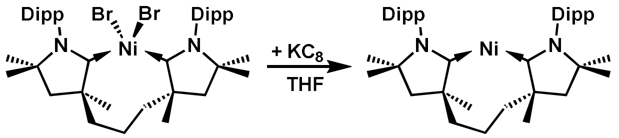

7 **8**

In an argon glovebox, a 25 mL round bottom flask containing a stir bar was charged with **7** (0.355 g, 0.443 mmol) and KC_8 (0.180 g, 1.33 mmol). The mixture was attached to a swivel frit and transferred to the vacuum line where it was cooled to −78 °C. THF (15 mL) was subsequently vacuum transferred on top of the solids and the mixture was allowed to warm up to room temperature over 30 minutes. The dark magenta solution was then stirred at room temperature for an additional 30 minutes, after which the volatiles were removed in vacuo. The residue was extracted with pentane (5 × 10 mL), the solution was filtered, and the solvent was removed in vacuo to yield **8** (0.186 g, 0.290 mmol, 65 %) as a microcrystalline black powder. X-ray quality crystals of **8** were obtained *via* slow evaporation of a pentane solution. Anal. Calcd. for $C_{41}H_{62}N_2Ni$: C 76.75; H 9.74; N 4.37. Found: C 76.59; H 9.69; N 4.34. **^{1}H NMR** (THF-d_8, 25°C, 400 MHz): δ 0.95 (d, $^3J_{HH}$ = 6.7 Hz, 3H, CH(C$\boldsymbol{H}_3$)$_2$) 1.09 (d, $^3J_{HH}$ = 6.8 Hz, 3H, CH(C$\boldsymbol{H}_3$)$_2$), 1.10 (s, 3H, C$\boldsymbol{H}_3$), 1.14 (s, 3H, C(C$\boldsymbol{H}_3$)$_2$), 1.23 (s, 3H, C(C$\boldsymbol{H}_3$)$_2$), 1.26 (d, $^3J_{HH}$ = 6.8 Hz, 3H, CH(C$\boldsymbol{H}_3$)$_2$), 1.72 (d, $^3J_{HH}$ = 6.8 Hz, 3H, CH(C$\boldsymbol{H}_3$)$_2$), 1.87 (s, 2H C$\boldsymbol{H}_2$), 2.91 (sept, $^3J_{HH}$ = 6.8 Hz, 1H, C$\boldsymbol{H}$(CH$_3$)$_2$), 3.16 (sept, $^3J_{HH}$ = 6.7 Hz, 1H, C$\boldsymbol{H}$(CH$_3$)$_2$), 6.90 (dd, $^3J_{HH}$ = 7.5 Hz, $^3J_{HH}$ = 1.4 Hz, 1H, *m*-C$_6\boldsymbol{H}_3$), 7.00 (dd, $^3J_{HH}$ = 7.5 Hz, $^3J_{HH}$ = 1.4 Hz, 1H, *m*-C$_6\boldsymbol{H}_3$)7.39 (vt, J = 7.5 Hz, 1H, *p*-C$_6\boldsymbol{H}_3$). The highly fluxional signals of the propyl linker were resolved at low temperature. **^{1}H NMR** (THF-d_8, −88°C, 400 MHz): δ 1.44 (m, 1H, CC$\boldsymbol{H}_2$CH$_2$), 1.62 (m, 2H, CC$\boldsymbol{H}_2$C$\boldsymbol{H}_2$), 2.07 (m, 1H, CC$\boldsymbol{H}_2$CH$_2$), 2.94 (m, 1H, CC$\boldsymbol{H}_2$CH$_2$), 3.33 (m, 1H, CCH$_2$C$\boldsymbol{H}_2$). **^{13}C NMR** (THF-d_8, −88 °C, 101 MHz): δ 20.7 (s,

CCH$_2$***C***H$_2$), 22.7, 22.8, 22.9, 23.7, 25.9, 26.0, 26.7, 27.6, 28.3, 28.7 (s, ***C***H(CH$_3$)$_2$), 28.8, 28.9, 29.3 (s, ***C***H(CH$_3$)$_2$), 30.1 (s, ***C***H(CH$_3$)$_2$), 30.4 (s, ***C***H(CH$_3$)$_2$), 30.5, 45.7 (s, CCH$_2$CH$_2$), 47.2 (s, ***C***H$_2$), 54.8 (s, ***C***H$_2$), 60.2 (s, ***C***(CH$_3$)CH$_2$), 60.6 (s, ***C***(CH$_3$)CH$_2$), 68.2, 74.6 (s, ***C***(CH$_3$)$_2$), 75.7 (s, ***C***(CH$_3$)$_2$), 124.0 (s, *m*-***C***$_6$H$_3$), 124.3 (s, *m*-***C***$_6$H$_3$), 124.5 (s, *m*-***C***$_6$H$_3$), 124.5 (s, *m*-***C***$_6$H$_3$), 127.6 (s, *p*-***C***$_6$H$_3$), 128.1 (s, *p*-***C***$_6$H$_3$), 138.6 (s, *i*-***C***$_6$H$_3$), 139.0 (s, *i*-***C***$_6$H$_3$), 145.1 (s, *o*-***C***$_6$H$_3$), 145.6 (s, *o*-***C***$_6$H$_3$), 145.7 (s, *o*-***C***$_6$H$_3$), 146.1 (s, *o*-***C***$_6$H$_3$), 227.2 (s, ***C***$_{carbene}$), 234.4 (s, ***C***$_{carbene}$).

Synthesis of nickel(I) complex 9

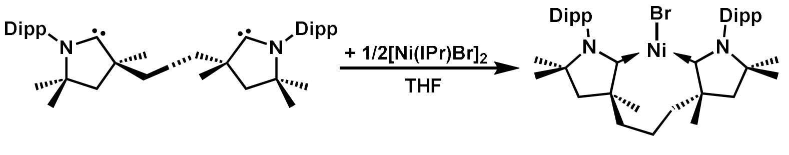

In an argon glovebox, a 25 mL round bottom flask containing a stir bar was charged with **3** (0.163 g, 0.278 mmol) and Sigman's bromide dimer (0.146 g, 0.139 mmol). Toluene (8 mL) was vacuum transferred on top of the solids and the solution was stirred overnight. The brown mixture was subsequently transferred to a swivel frit and filtered on the vacuum line. The solid was washed with pentane (5 mL) and dried under vacuum to yield **9** (0.137 g, 0.190 mmol, 69 %) as a maroon, microcrystalline solid. X-ray quality crystals were obtained *via* slow diffusion of pentane into a THF solution of **9**. Magnetic susceptibility μ_{eff} was determined to be 2.20 (1 unpaired electron) at 298 K using Evans method. The chemical shift of Si(SiMe$_3$)$_4$ and a 0.014 M solution of **9** in CD_2Cl_2 were used. Anal. Calcd. for $C_{41}H_{62}N_2BrNi$: C 68.25; H 8.66; N 3.88. Found: C 68.33; H 9.01; N 3.91. HRMS (ESI) m/z: $[M+H]^+$ Calcd. for $C_{41}H_{62}N_2BrNi$ 640.4289; Found: 640.4261.

NMR Spectra

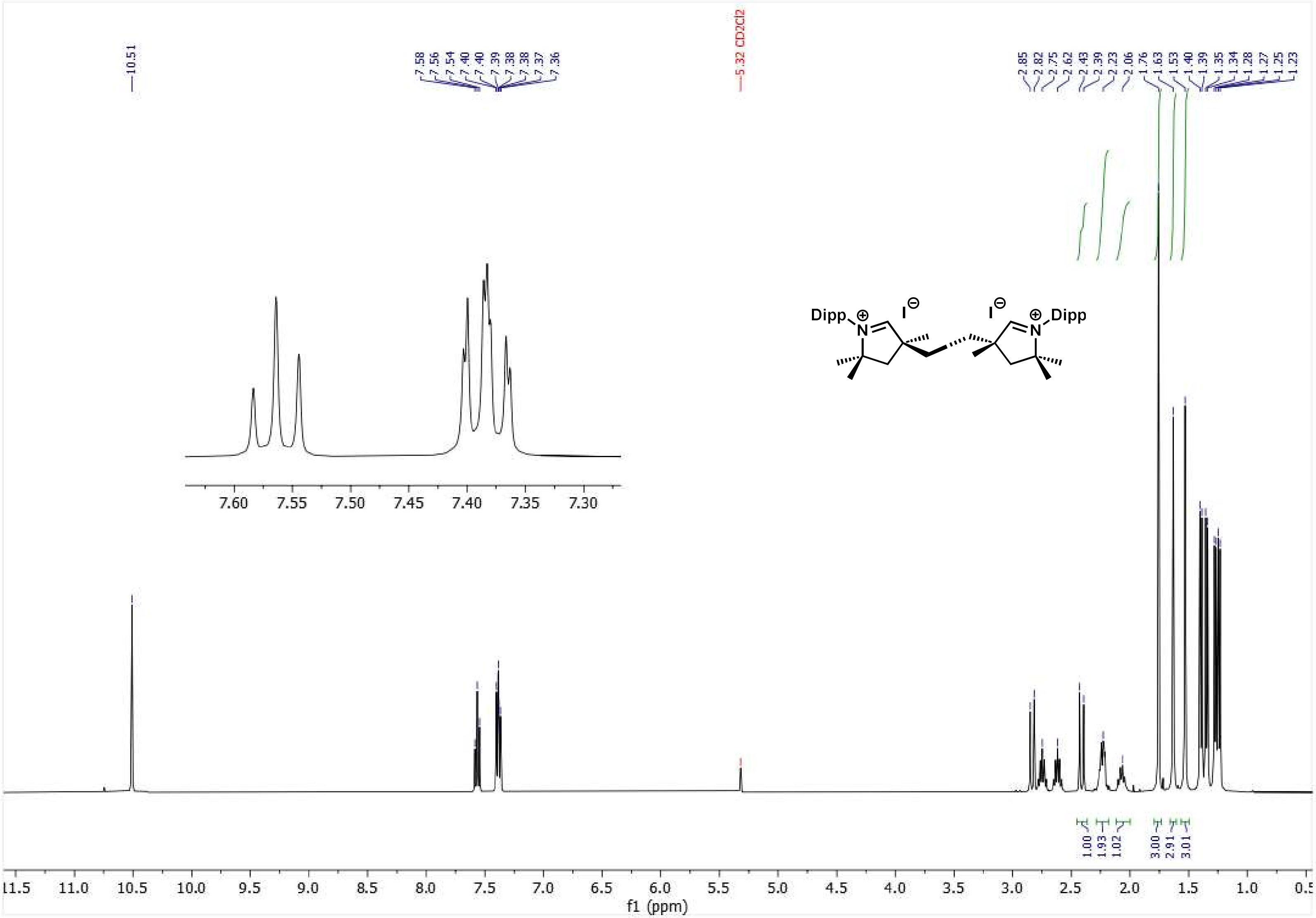

Supplementary Fig. 1. ^{1}H NMR spectrum of **2** (400 MHz, 298 K, CD_2Cl_2)

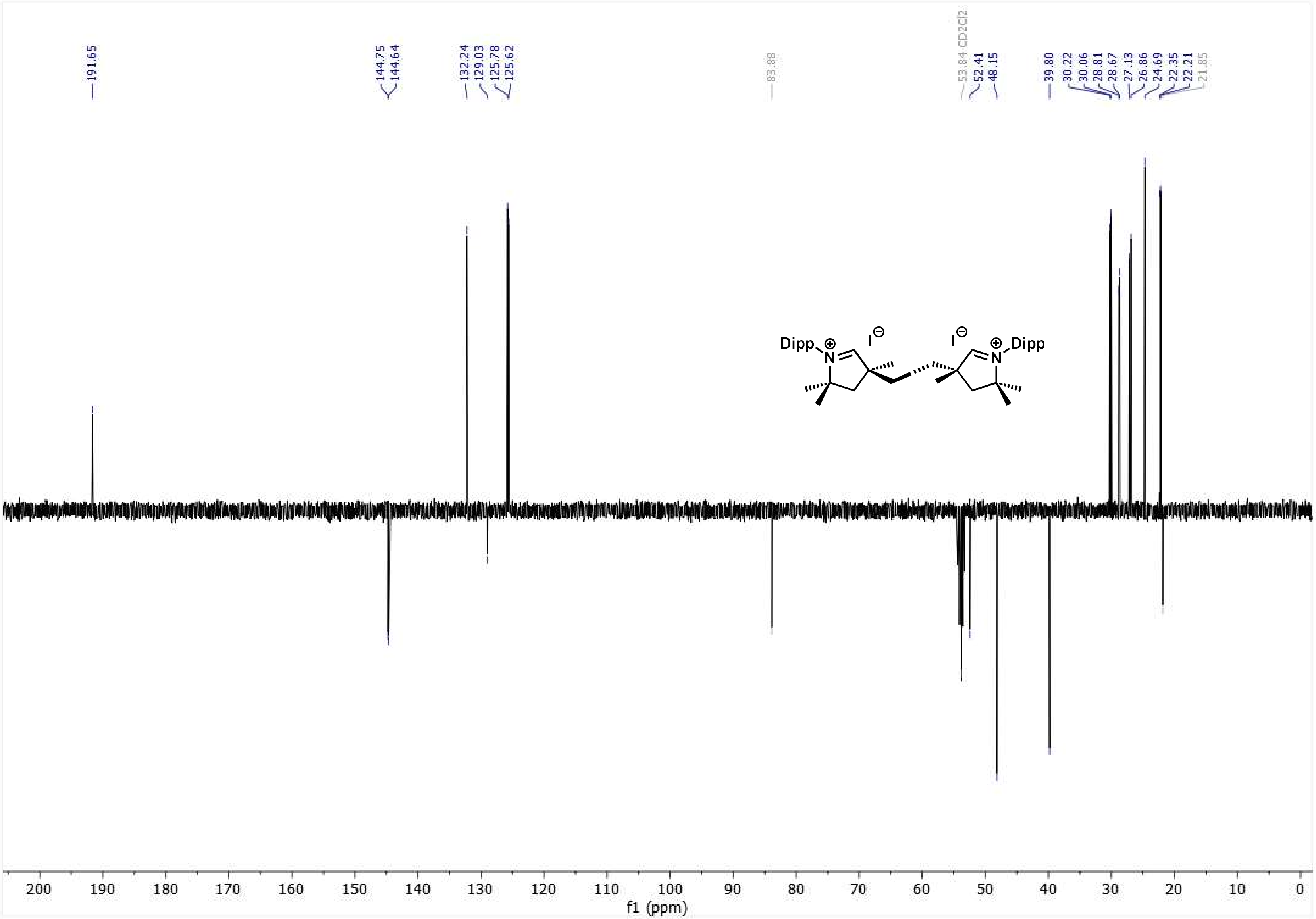

Supplementary Fig. 2. ^{13}C DEPTQ NMR spectrum of **2** (101 MHz, 298 K, CD_2Cl_2).

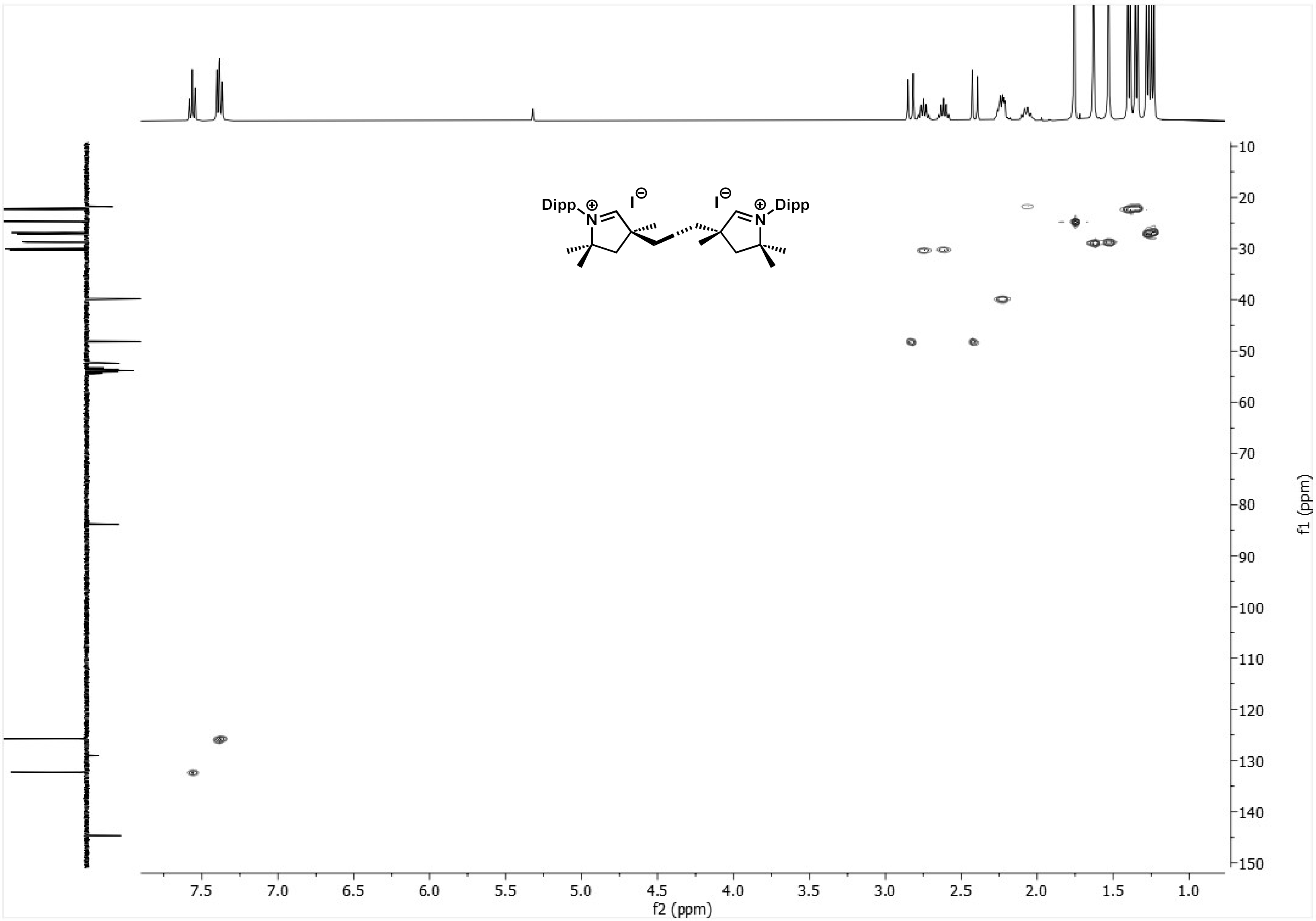

Supplementary Fig. 3. ^{1}H-^{13}C HSQC NMR spectrum of **2** (400MHz, 298 K, CD_2Cl_2).

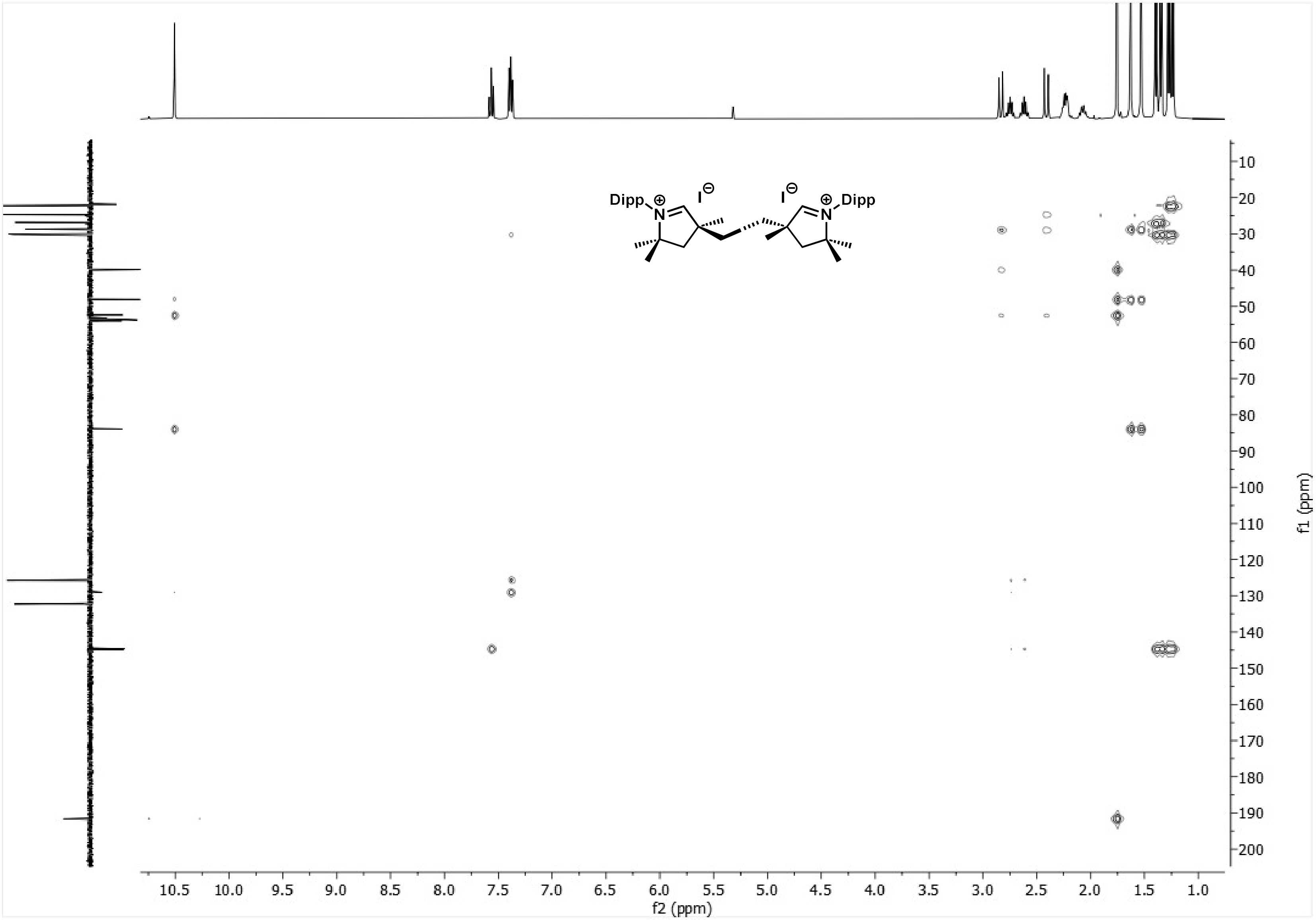

Supplementary Fig. 4. ^{1}H-^{13}C HMBC NMR spectrum of **2** (400 MHz, 298 K, CD_2Cl_2).

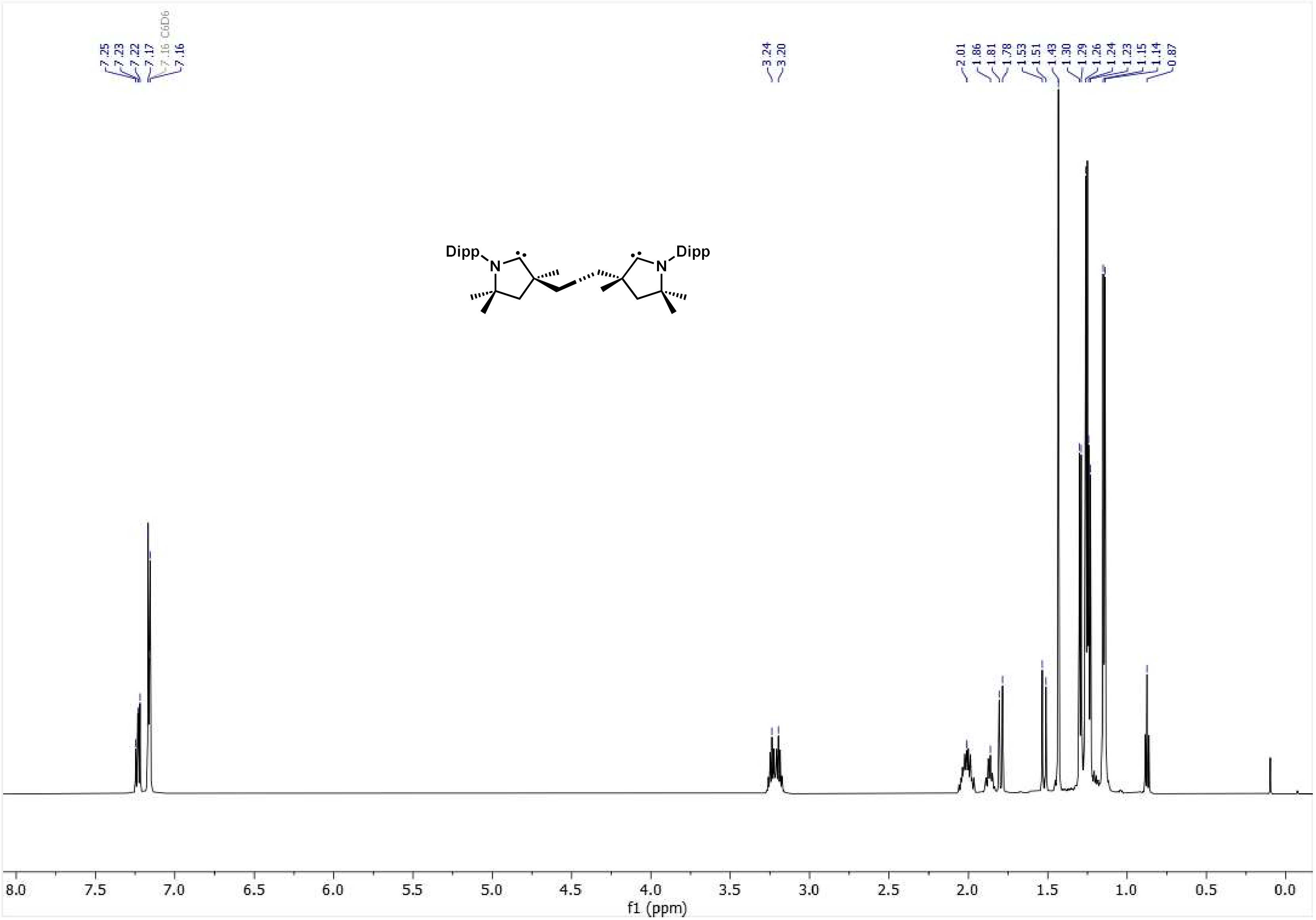

Supplementary Fig. 5. ^{1}H NMR spectrum of **3** (400 MHz, 298 K, C_6D_6).

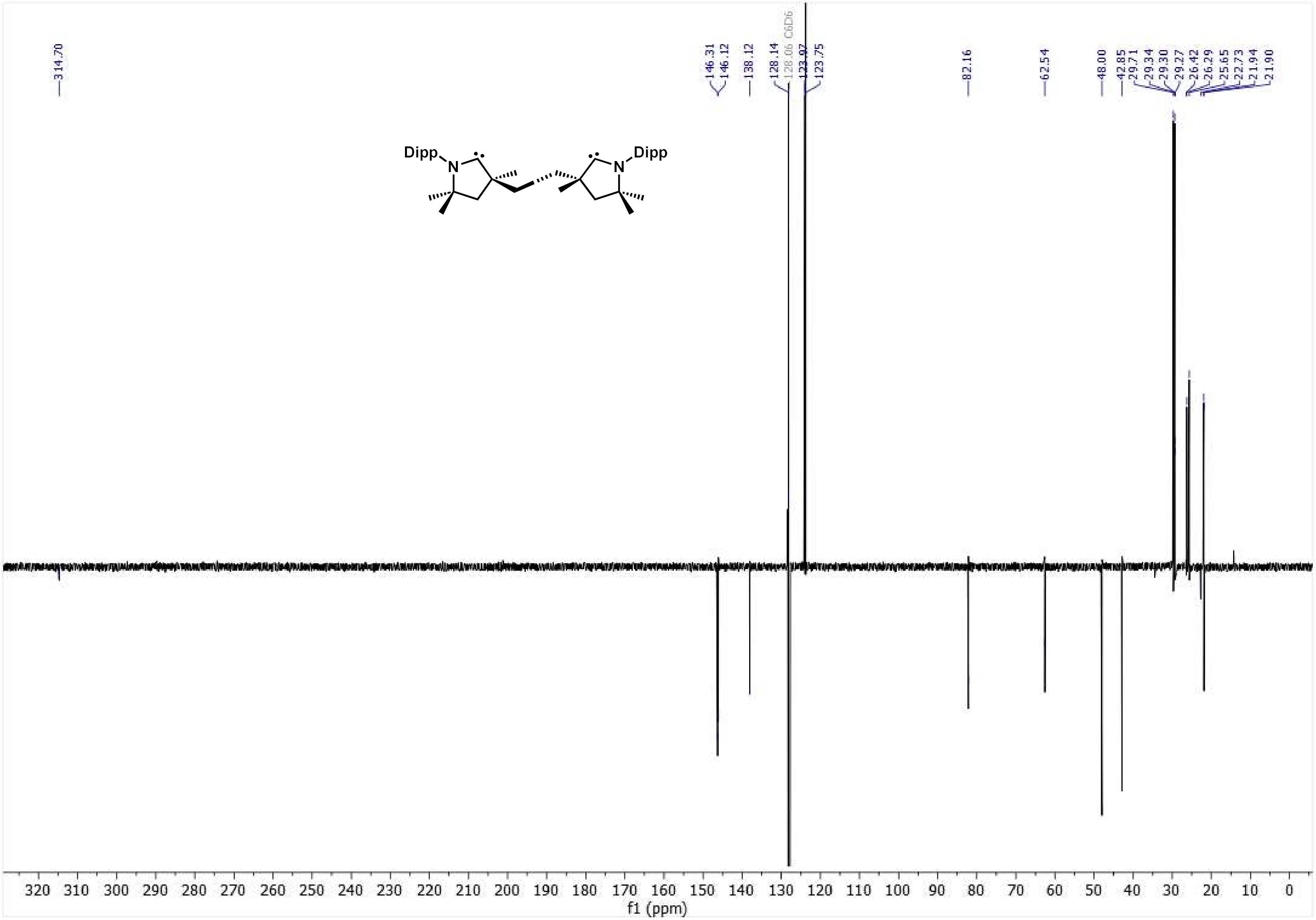

Supplementary Fig. 6. ^{13}C DEPTQ NMR spectrum of **3** (101 MHz, 298 K, C_6D_6).

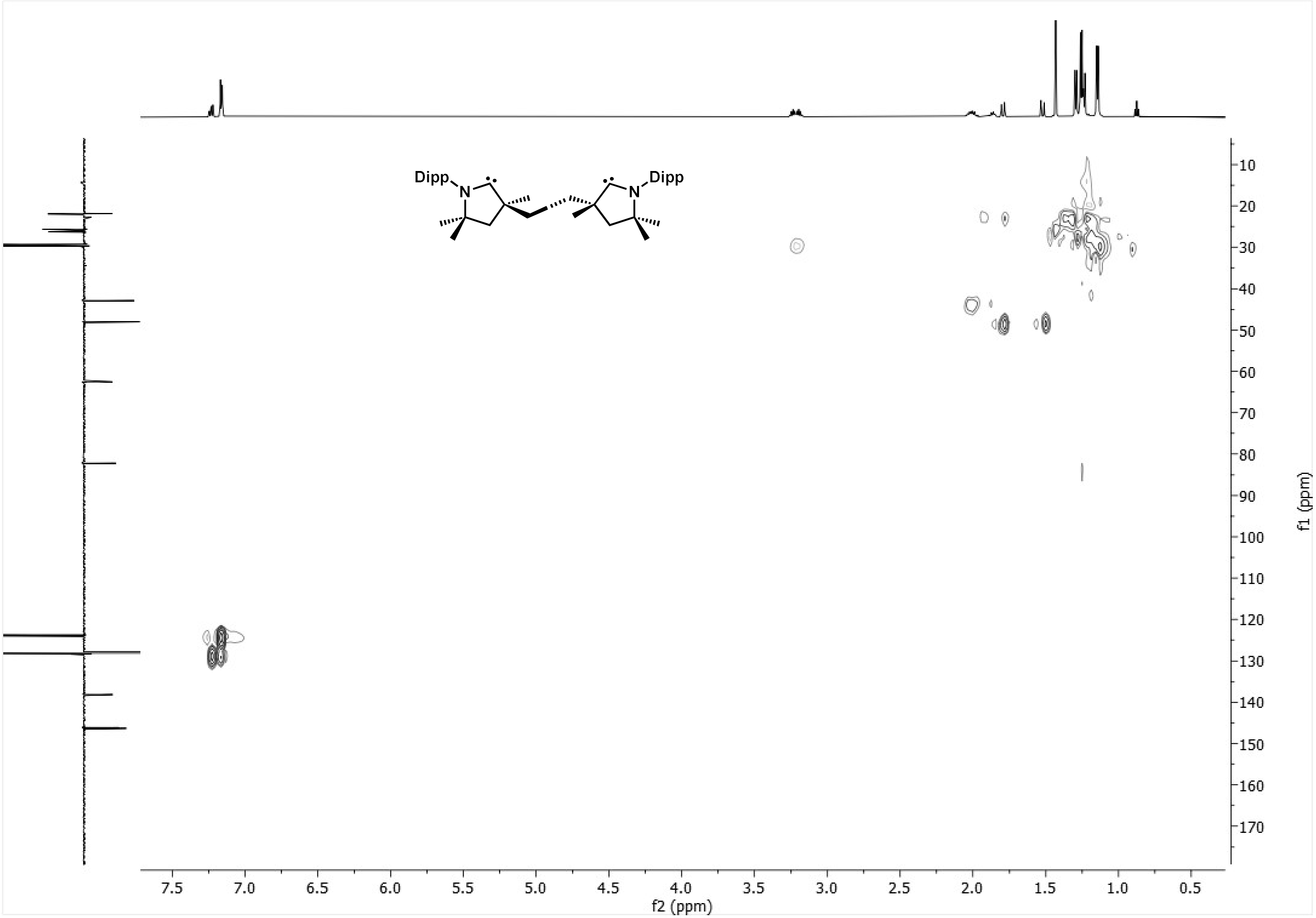

Supplementary Fig. 7. ^{1}H-^{13}C HSQC spectrum of **3** (400 MHz, 298 K, C_6D_6).

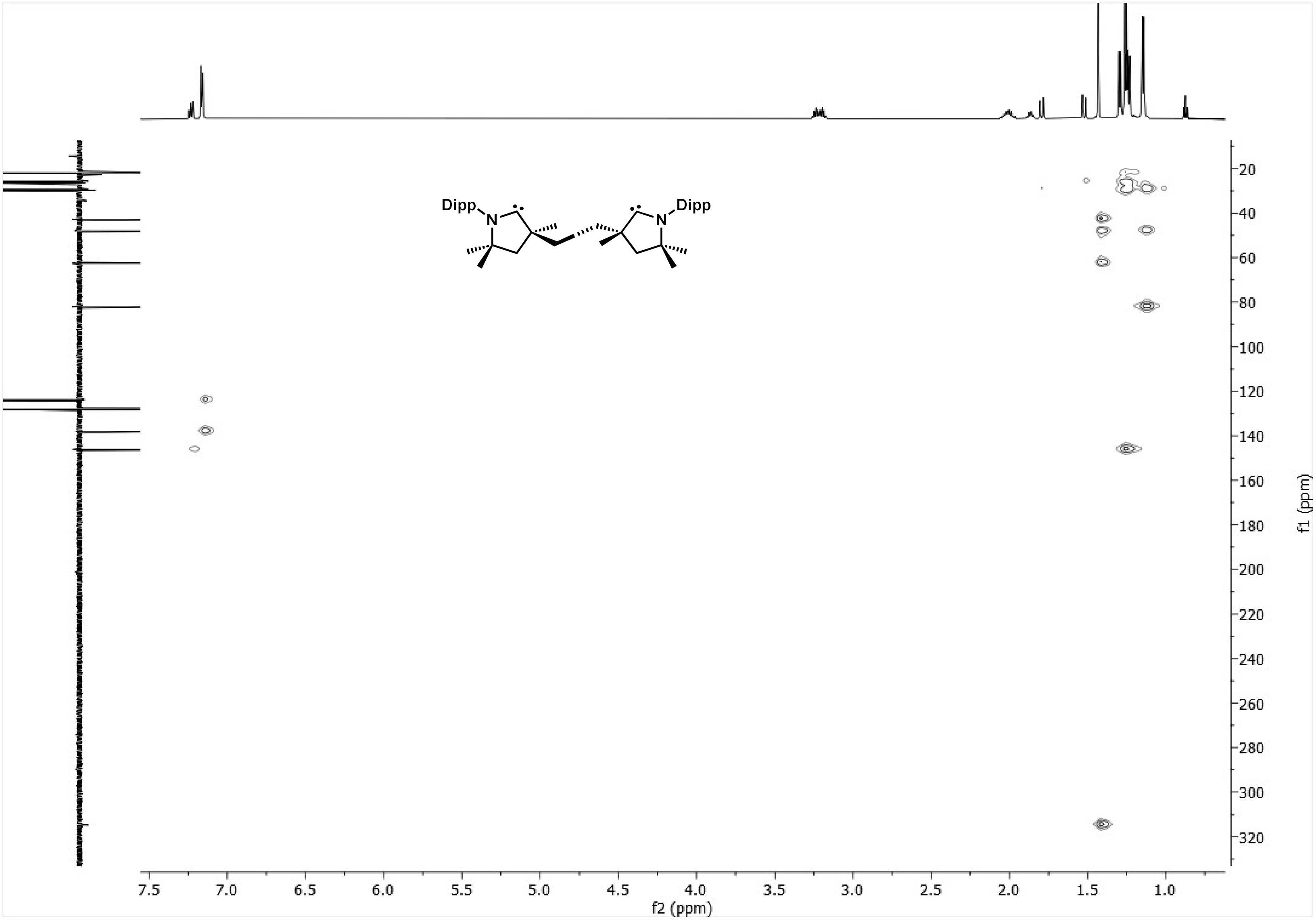

Supplementary Fig. 8. ^{1}H-^{13}C HMBC spectrum of **3** (400 MHz, 298 K, C_6D_6).

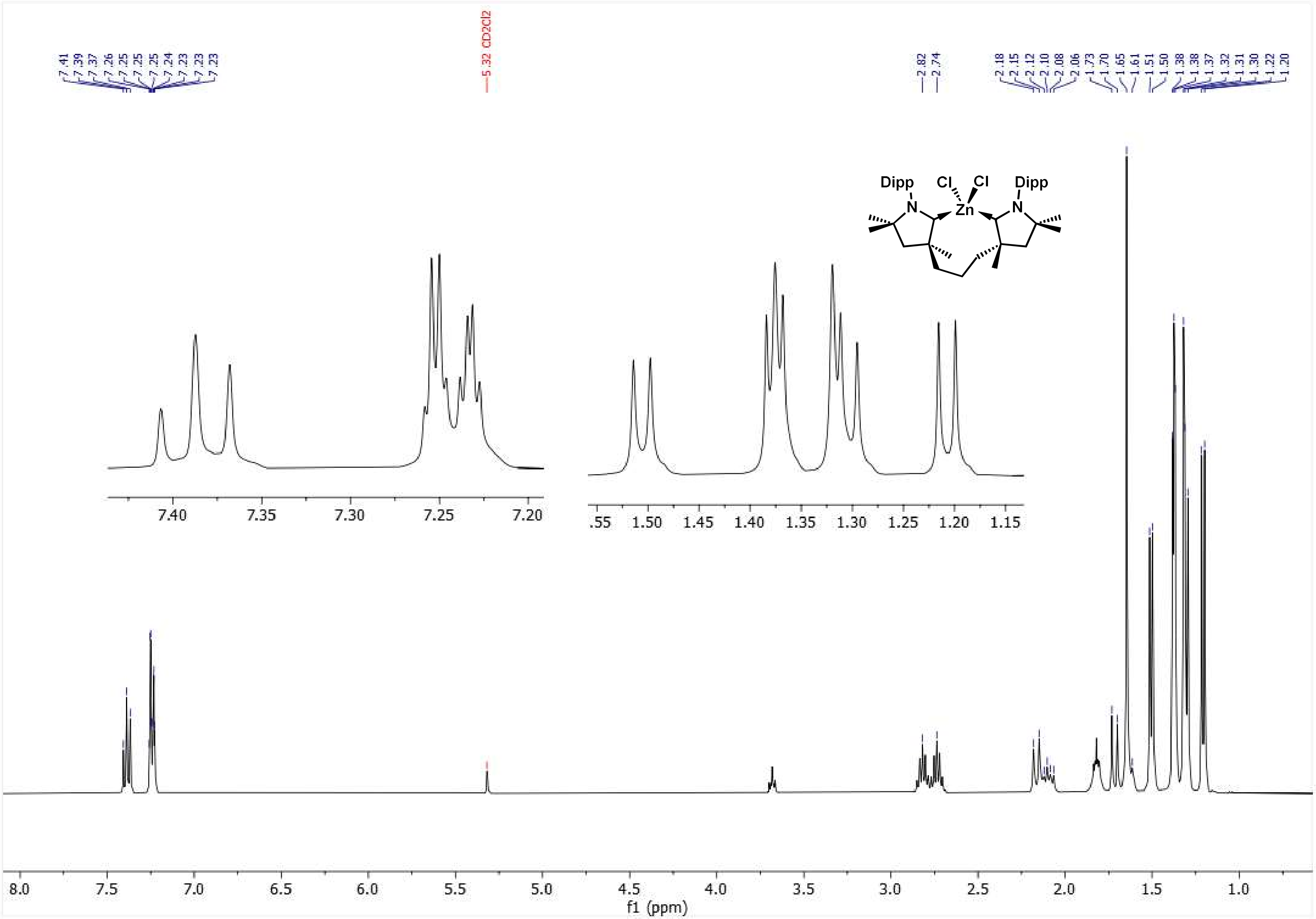

Supplementary Fig. 9. ^{1}H NMR spectrum of **6** (400 MHz, 298 K, CD_2Cl_2)

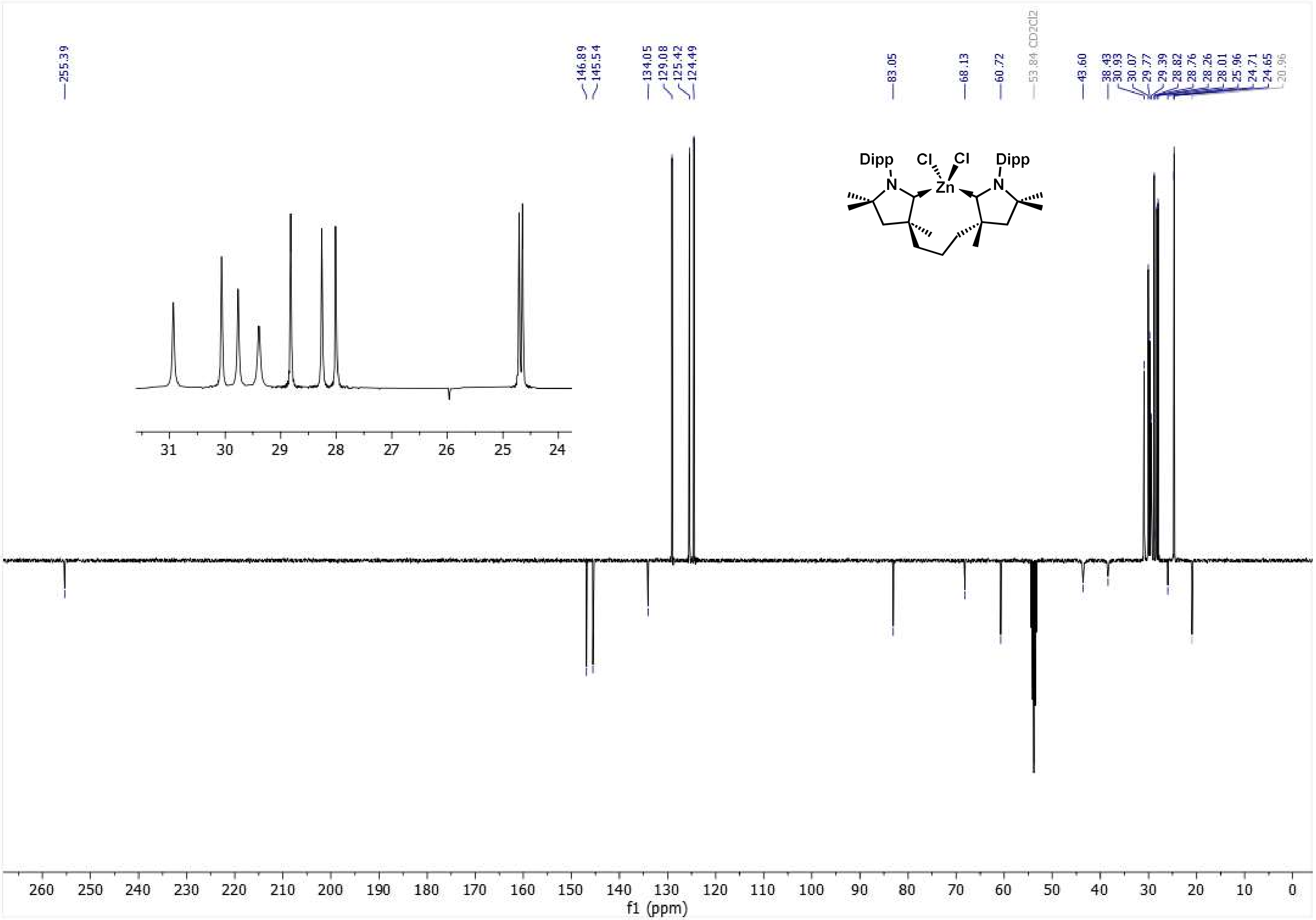

Supplementary Fig. 10. ^{13}C NMR spectrum of **6** (101 MHz, 298 K, CD_2Cl_2).

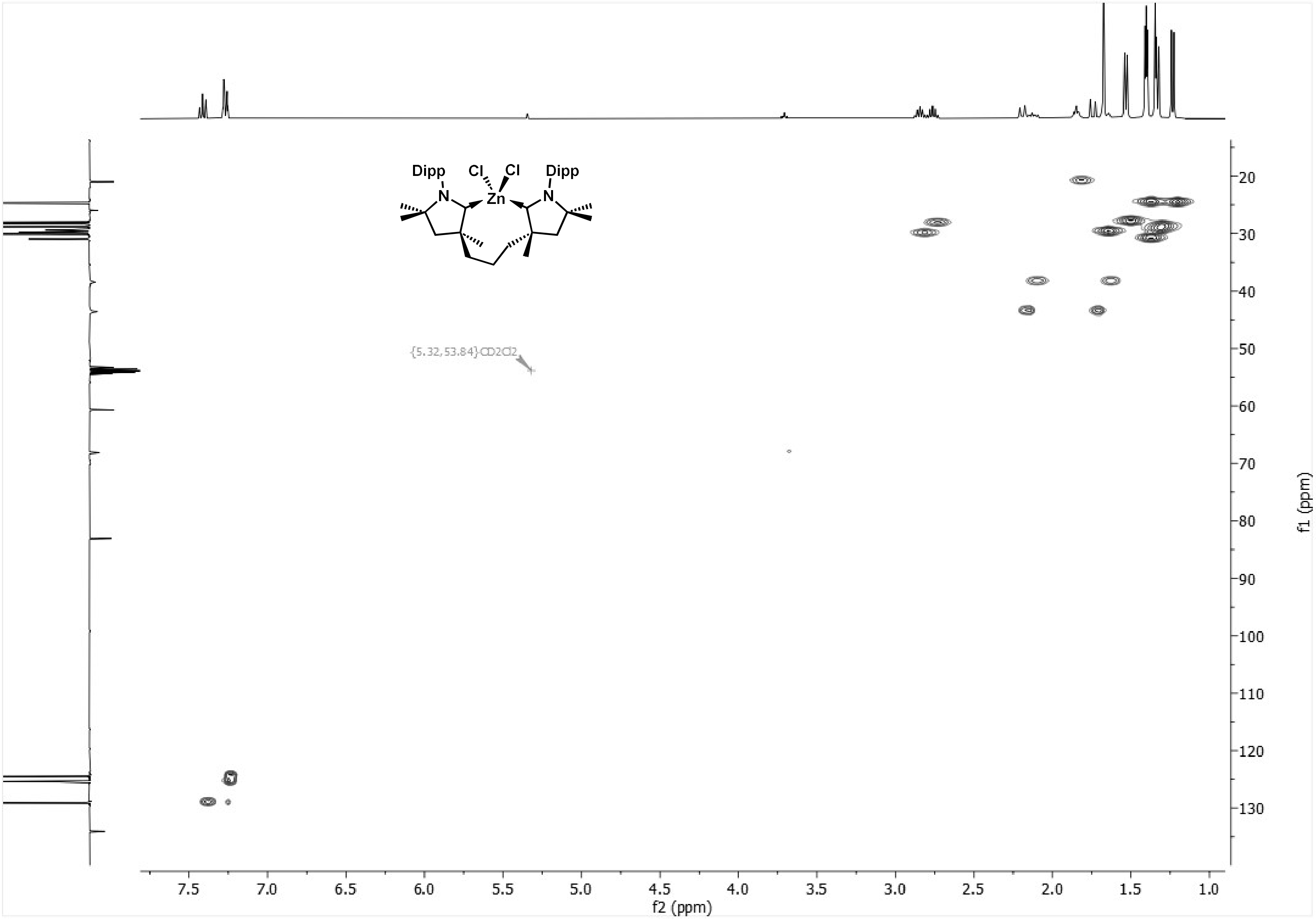

Supplementary Fig. 11. ^{1}H-^{13}C HSQC NMR spectrum of **6** (400MHz, 298 K, CD_2Cl_2).

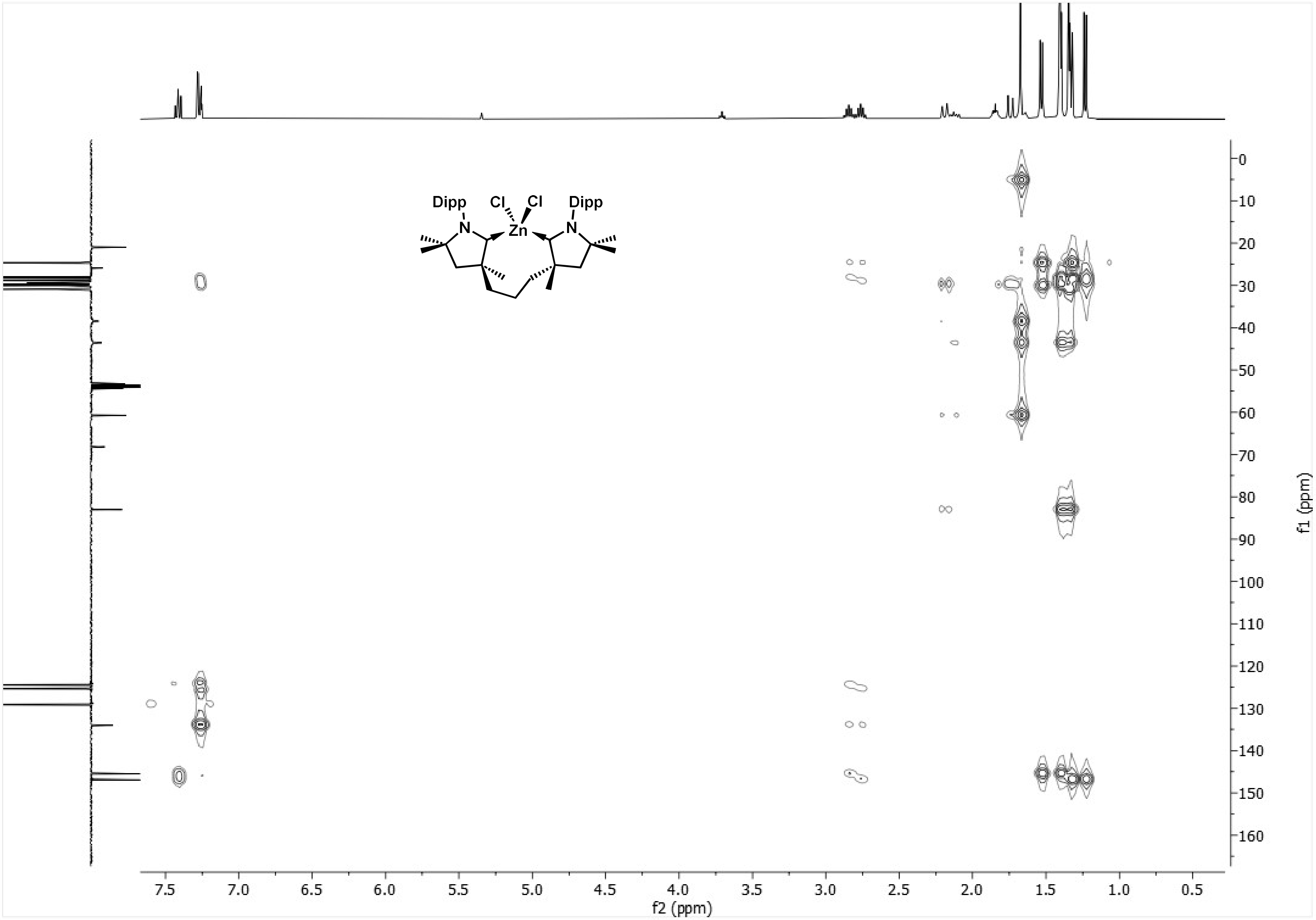

Supplementary Fig. 12. ^{1}H-^{13}C HMBC NMR spectrum of **6** (400MHz, 298 K, CD_2Cl_2)

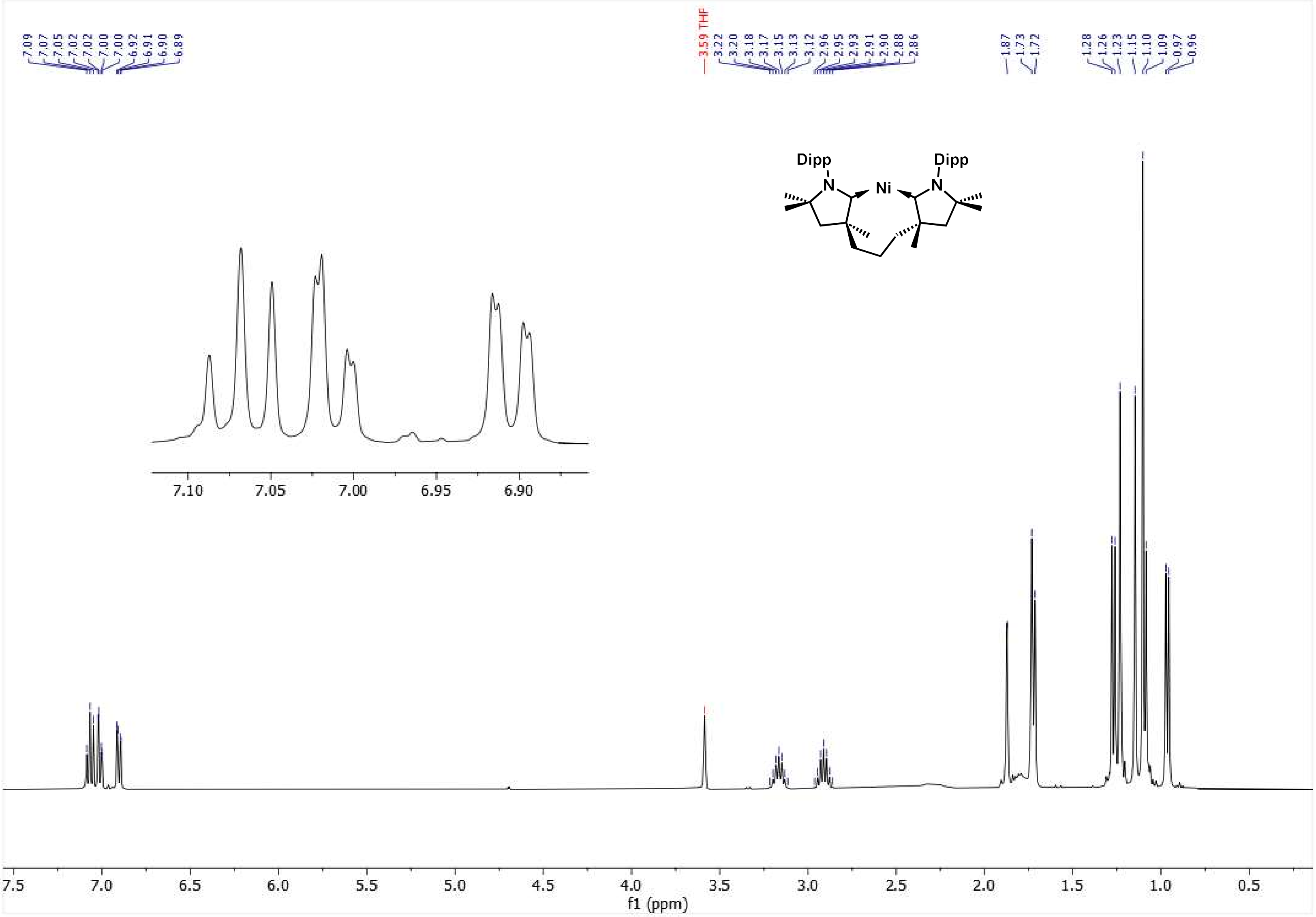

Supplementary Fig. 13. ^{1}H NMR spectrum of **8** (400 MHz, 298 K, THF-d_8).

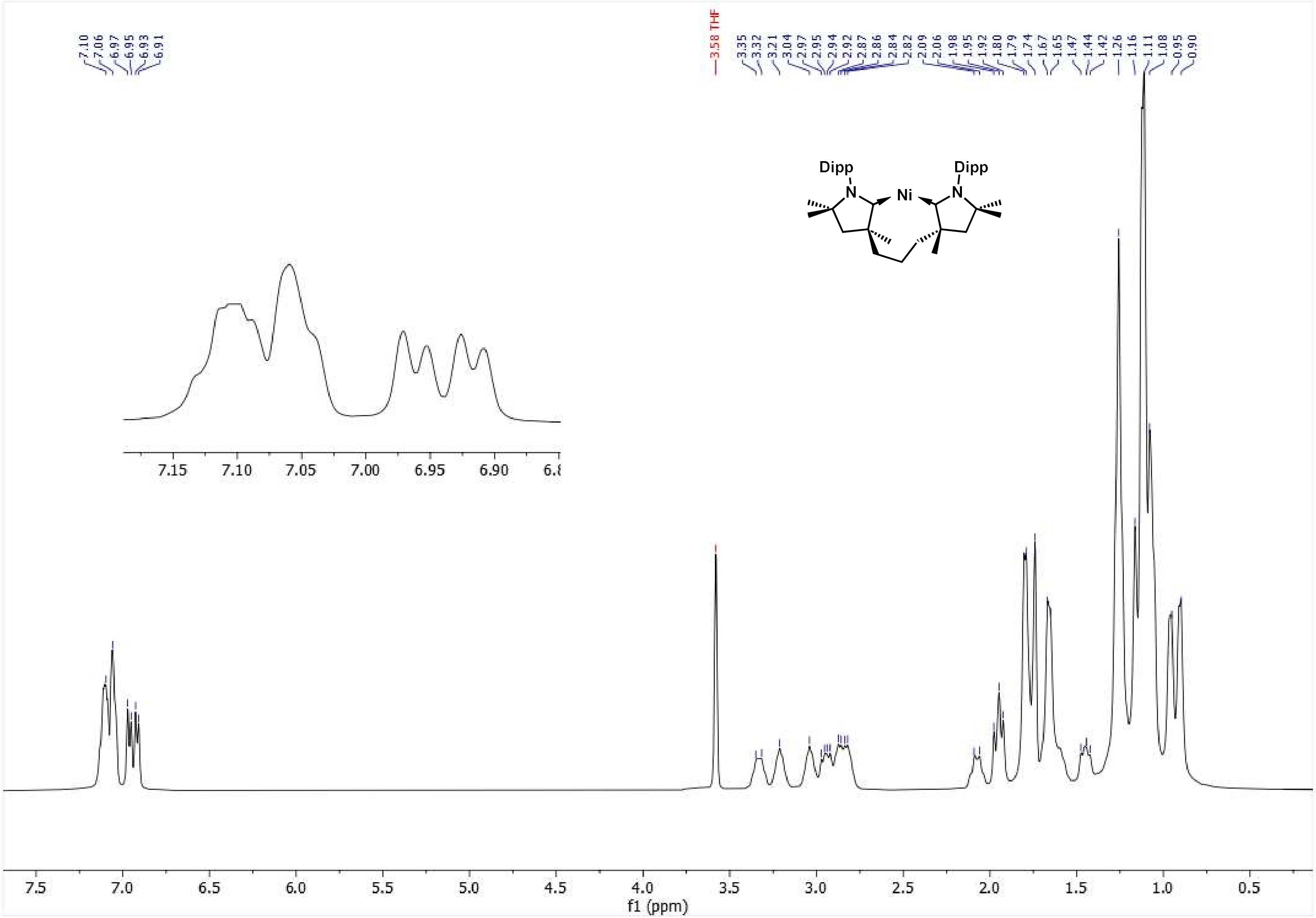

Supplementary Fig. 14. Low temperature ^{1}H NMR spectrum of **8** (400 MHz, 185 K, THF-d_8).

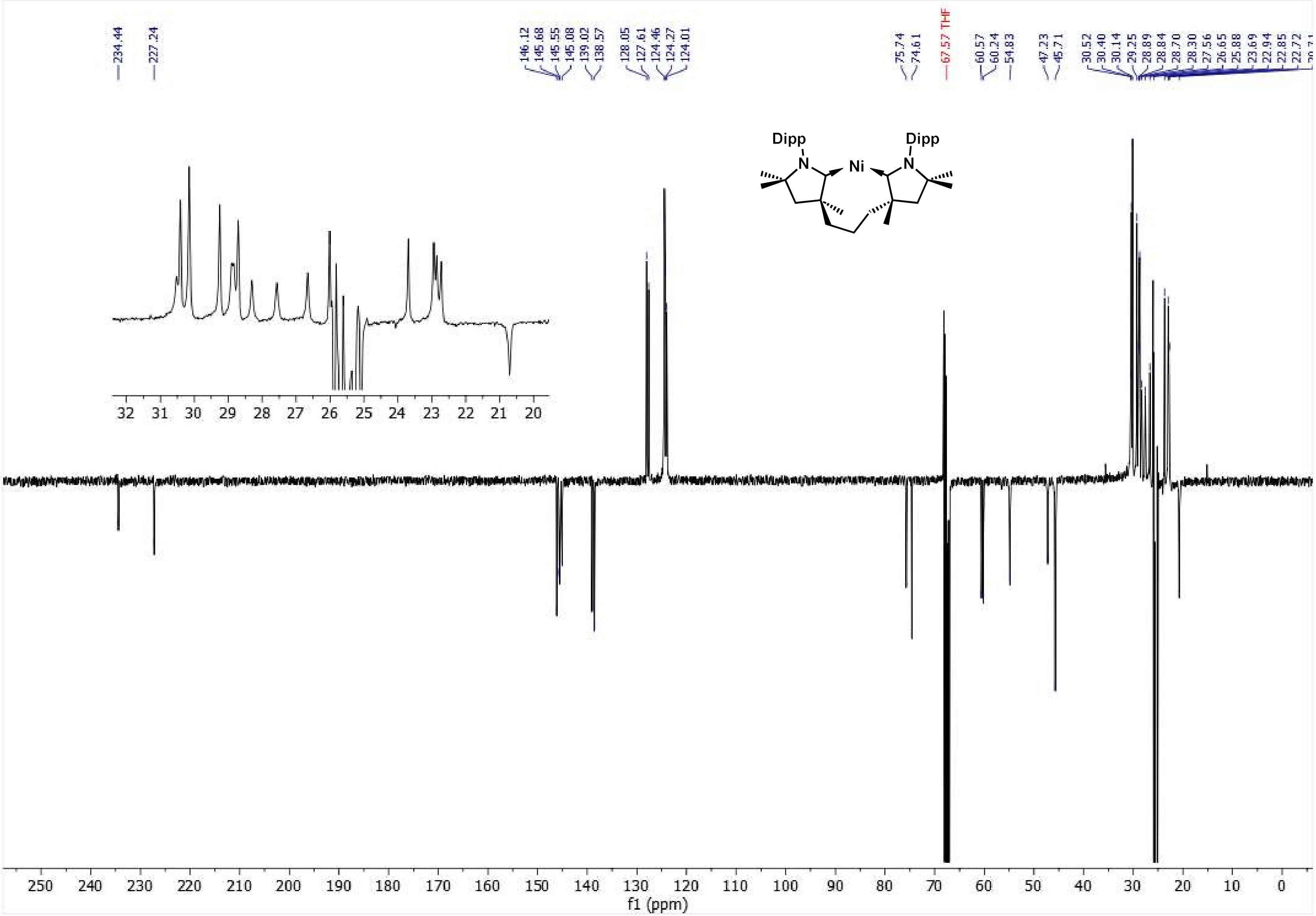

Supplementary Fig. 15. Low temperature ^{13}C DEPTQ NMR spectrum of **8** (400 MHz, 185 K, THF-d_8).

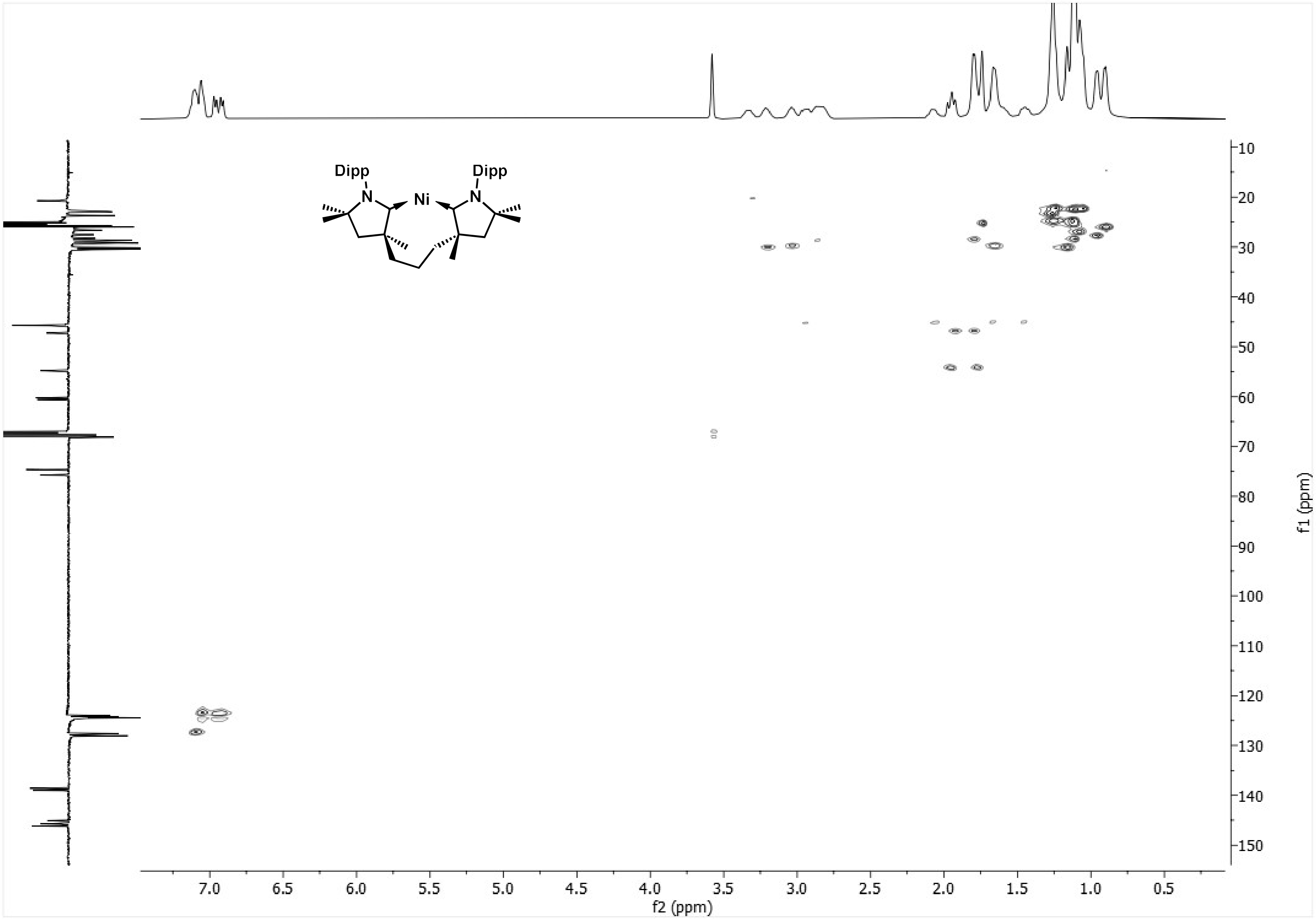

Supplementary Fig. 16. Low temperature ^{1}H-^{13}C HSQC spectrum of **8** (400 MHz, 185 K, THF-d_8).

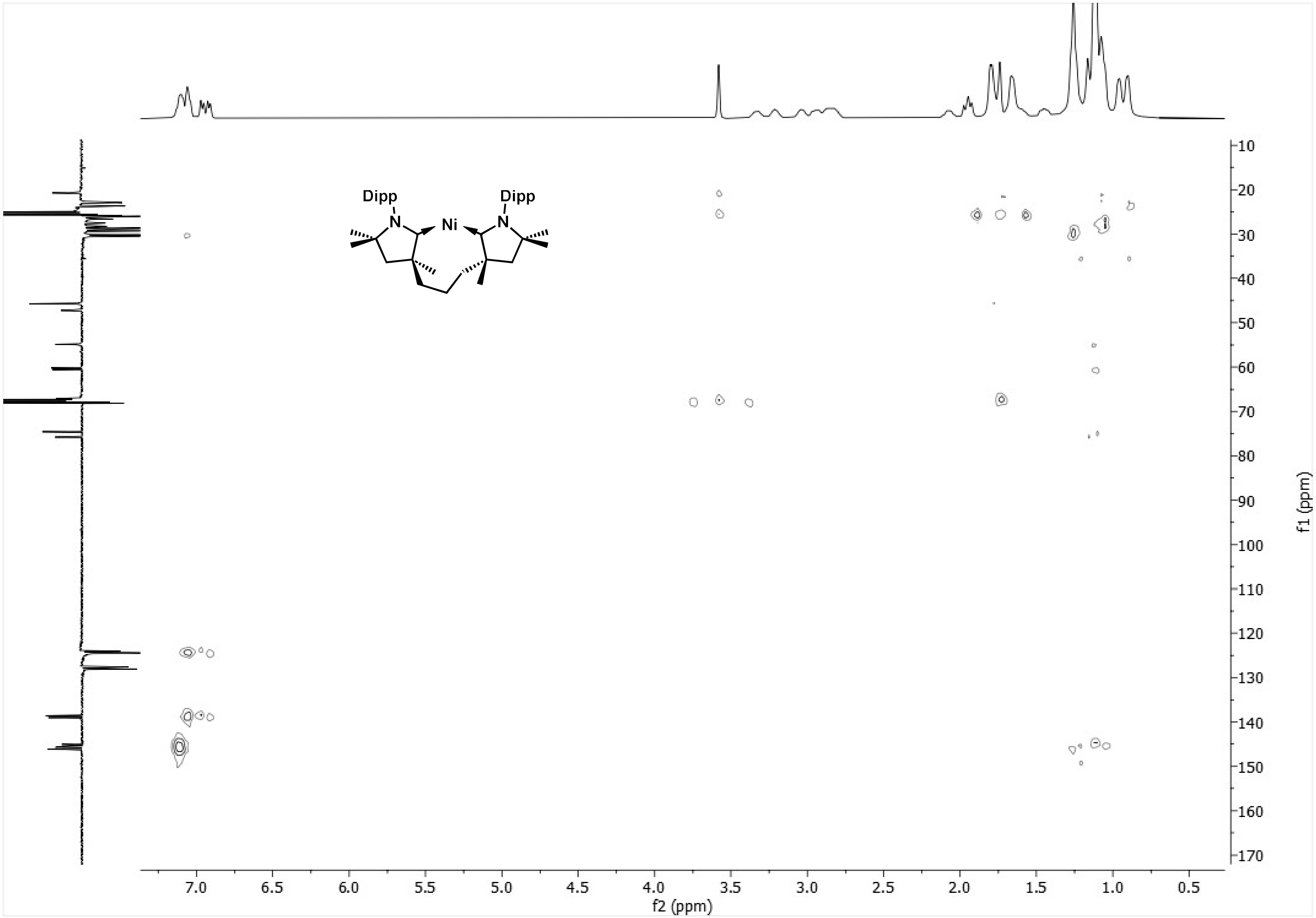

Supplementary Fig. 17. Low temperature ^{1}H-^{13}C HMBC spectrum of **8** (400 MHz, 185 K, THF-d_8).

Crystallographic Details

Supplementary Table 1. Summary of Crystallographic Data for Compounds **3** and **4–9**[a]

	**3**	**4**	**5**	**6**
CCDC Deposition #	2277362	2277363	2277364	2277365
Formula	$C_{41}H_{62}N_2$	$C_{41}H_{62}Cl_2FeN_2$	$C_{41}H_{62}Cl_2CoN_2$	$C_{41}H_{62}Cl_2ZnN_2$
Formula weight	582.93	709.68	712.76	719.19
Crystal system	Monoclinic	Monoclinic	Monoclinic	Monoclinic
Space group	$I2/a$	$P2_1/c$	$P2_1/c$	$P2_1/c$
a [Å]	15.3561(4)	13.2103(9)	13.1564(13)	13.2047(8)
b [Å]	13.7696(4)	17.4705(12)	17.4174(17)	17.4407(11)
c [Å]	18.3570(6)	17.2918(12)	17.3018(17)	17.3013(11)
α [°]	90	90	90	90
β [°]	107.708(2)	105.698(1)	105.878(1)	105.8140(10)
γ [°]	90	90	90	90
V [Å^3]	3697.63(19)	3841.9(5)	3813.4(6)	3833.7(4)
Z	4	4	4	4
T [K]	173	173	173	173
λ [Å]	1.54178	0.71073	0.71073	0.71073
ρ_{calc} [gcm^{-3}]	1.047	1.227	1.241	1.246
F(000)	1288.0	1528.0	1532.0	1544.0
R(int)	0.0284	0.0708	0.0686	0.0417
μ [mm^{-1}]	0.441	0.562	0.620	0.810
2θ range [°]	8.172 – 136.618	3.202 – 54.97	3.218 – 54.944	3.206 – 54.944
Total data	3370	56833	55397	57162
Unique	3370	8750	8687	8791
Compl. [%]	99.1	99.2	99.6	100
Parameters	202	429	429	429
R_1 (I>2σ(I))	0.0559	0.0393	0.0393	0.0336
wR_2 (all data)	0.1531	0.0947	0.0962	0.0866
GOF	1.078	1.003	1.020	1.027

Supplementary Table 1. Continued

	**7**	**8**[b]	**9**
CCDC Deposition #	2277366	2277367	2277368
Formula	$C_{41}H_{62}Br_2NiN_2$	$C_{41}H_{62}NiN_2$	$C_{41}H_{62}BrNiN_2$
Formula weight	801.45	641.62	721.54
Crystal system	Monoclinic	Triclinic	Monoclinic
Space group	$P2_1/c$	P-1	$C2/c$
a [Å]	13.1465(8)	10.3827(10)	9.758(2)
b [Å]	17.6602(10)	10.6758(10)	15.715(3)
c [Å]	17.2343(10)	18.3352(17)	24.531(5)
α [°]	90	96.475(5)	90
β [°]	104.4590(10)	100.896(5)	90.919(3)
γ [°]	90	107.504(5)	90
V [Å^3]	3875.0(4)	1871.8(3)	3761.1(14)
Z	4	2	4
T [K]	173	173	173
λ [Å]	0.71073	1.54178	0.71073
ρ_{calc} [gcm^{-3}]	1.374	1.138	1.274
F(000)	1680.0	700.0	1540.0
R(int)	0.0650	0.0608	0.0540
μ [mm^{-1}]	2.593	0.944	1.608
2θ range [°]	3.2 – 55.292	4.99 – 136.572	3.32 – 55.07
Total data	58094	21278	25943
Unique	8987	6537	4319
Compl. [%]	99.5	95.5	99.7
Parameters	429	411	212
R_1 (I>2σ(I))	0.0286	0.0568	0.0461
wR_2 (all data)	0.0705	0.1669	0.1027
GOF	1.024	1.074	1.105

[a] Deposition Numbers 2277362-2277368 contain the supplementary crystallographic data for this paper. These data are provided free of charge by the joint Cambridge Crystallographic Data Centre and Fachinformationszentrum Karlsruhe Access Structures service www.ccdc.cam.ac.uk/structures.

[b] A B-level alert referring to low data completion (95.5%) is due to a poorly diffracting crystal despite 80s/frame at high angle, in combination with limited stability of the crystal, and hardware-imposed restrictions of the theta angle range available to the detector during data collection.

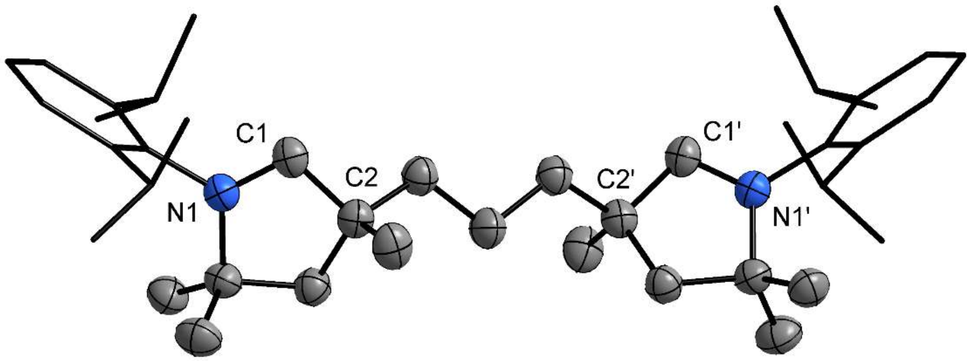

Supplementary Fig. 18. Solid-state structure of **3** with thermal ellipsoids drawn at 50 % probability and hydrogen atoms omitted for clarity. Selected bond lengths [Å] and angles [°]: C1-N1 1.312(2); C1-C2 1.526(3); N1-C1-C2 106.01(17).

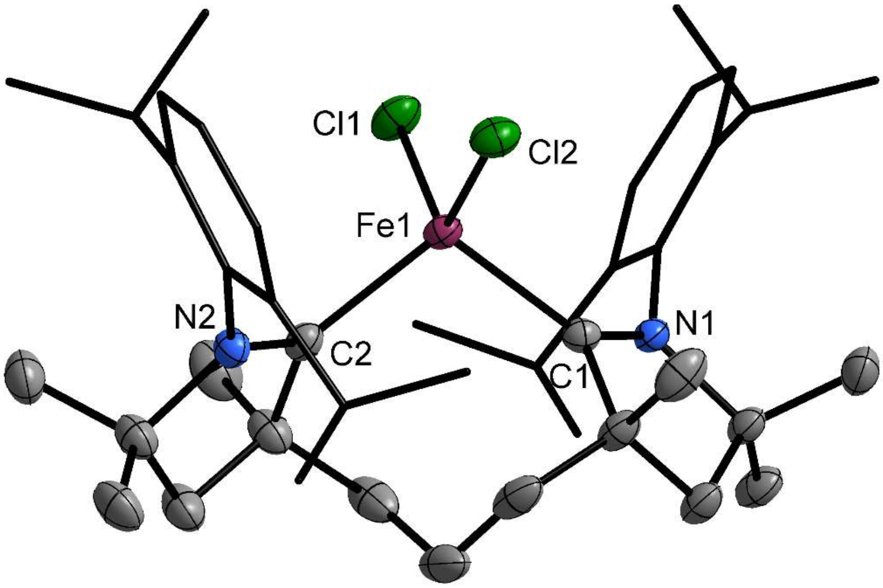

Supplementary Fig. 19. Solid-state structure of **4** with thermal ellipsoids drawn at 50 % probability and hydrogen atoms omitted for clarity. Selected bond lengths [Å] and angles [°]: Fe1-C1 2.1293(19); Fe1-C2 2.139(2); Fe1-Cl1 2.2848(6); Fe1-Cl2 2.2857(6); C1-N1 1.315(2); C2-N2 1.318(2); C1-Fe1-C2 107.68(7); Cl1-Fe1-Cl2 114.68(2).

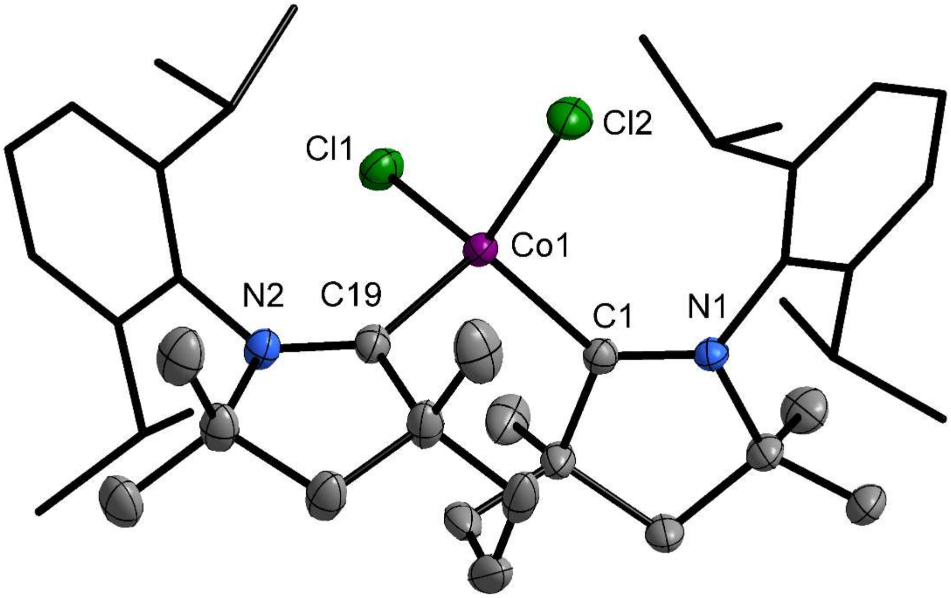

Supplementary Fig. 20. Solid-state structure of **5** with thermal ellipsoids drawn at 50 % probability and hydrogen atoms omitted for clarity. Selected bond lengths [Å] and angles [°]: Co1-C1 2.091(2); Co1-C19 2.097(2); Co1-Cl1 2.2645(6); Co1-Cl2 2.2668(6); C1-N1 1.310(2); C19-N2 1.311(3); C1-Co1-C19 109.98(8); Cl1-Co1-Cl2 112.01(2).

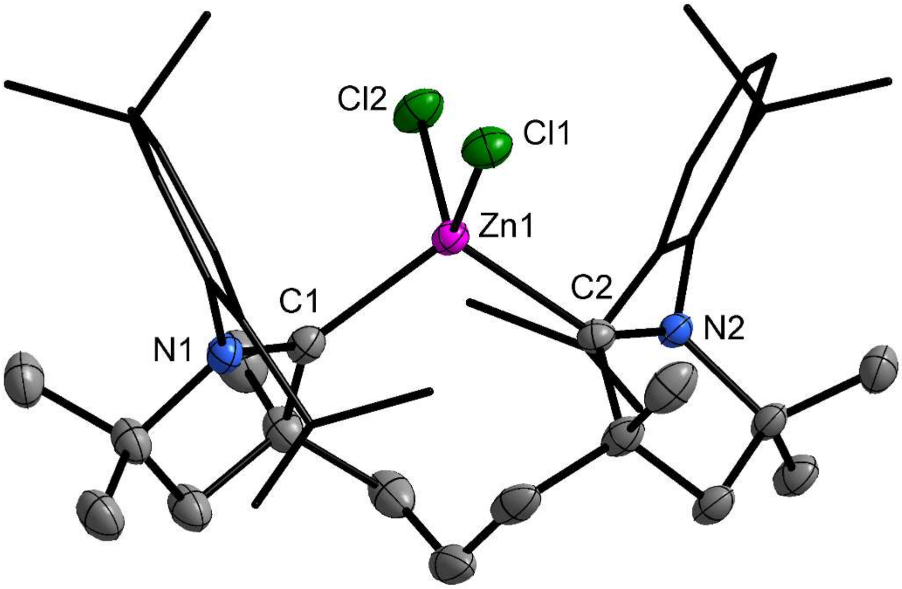

Supplementary Fig. 21. Solid-state structure of **6** with thermal ellipsoids drawn at 50 % probability and hydrogen atoms omitted for clarity. Selected bond lengths [Å] and angles [°]: Zn1-C1 2.1302(18); Zn1-C2 2.1265(16); Zn1-Cl1 2.2873(5); Zn1-Cl2 2.2898(5); C1-N1 1.309(2); C2-N2 1.307(2); C1-Zn1-C2 109.98(6); Cl1-Zn1-Cl2 111.458(19).

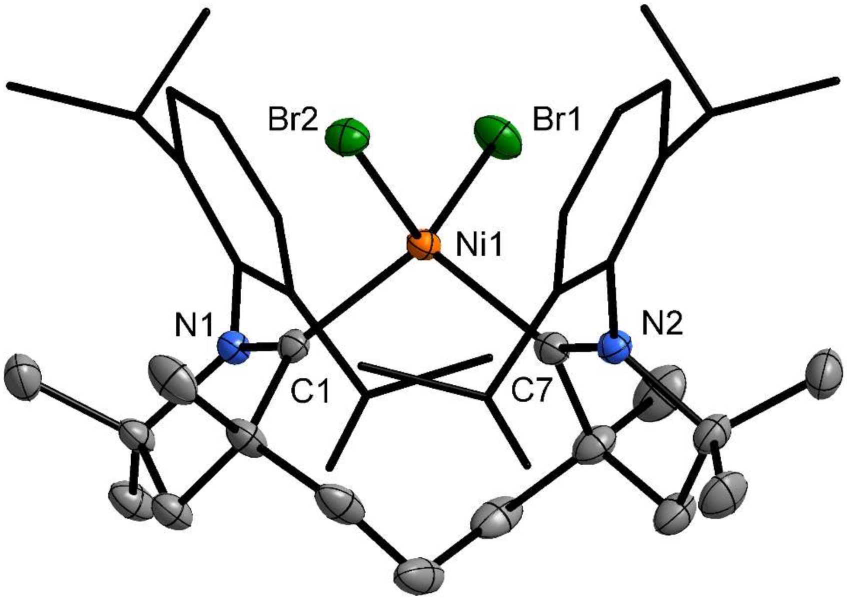

Supplementary Fig. 22. Solid-state structure of **7** with thermal ellipsoids drawn at 50 % probability and hydrogen atoms omitted for clarity. Selected bond lengths [Å] and angles [°]: Ni1-C1 2.0491(18); Ni1-C7 2.0528(19); Ni1-Br1 2.404(3); Ni1-Br2 2.403(3); C1-N1 1.314(2); C7-N2 1.313(2); C1-Ni1-C7 107.72(7); Br1-Ni1-Br2 116.443(11).

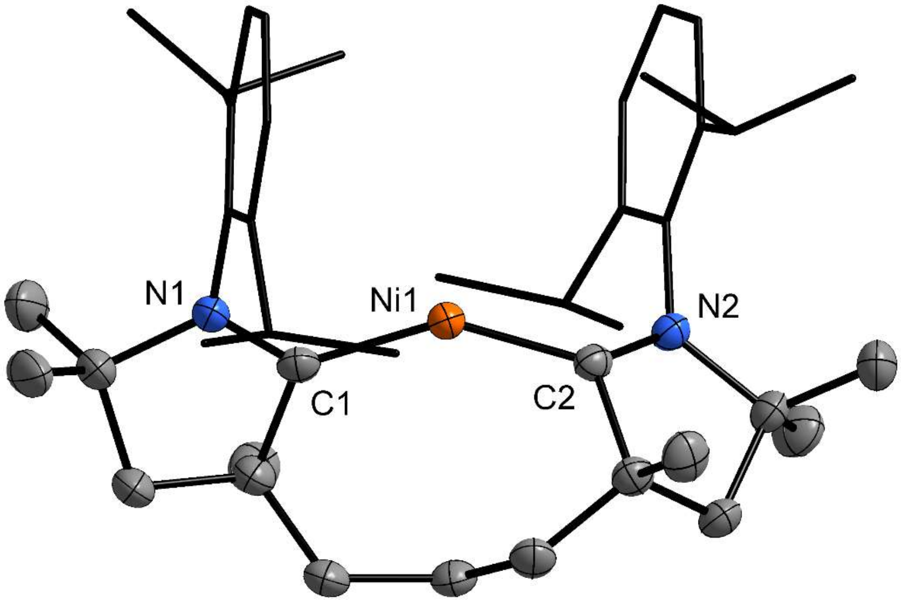

Supplementary Fig. 23. Solid-state structure of **8** with thermal ellipsoids drawn at 50 % probability and hydrogen atoms omitted for clarity. Selected bond lengths [Å] and angles [°]: Ni1-C1 1.8209(18); Ni1-C2 1.8103(17); C1-Ni1-C2 146.70(8).

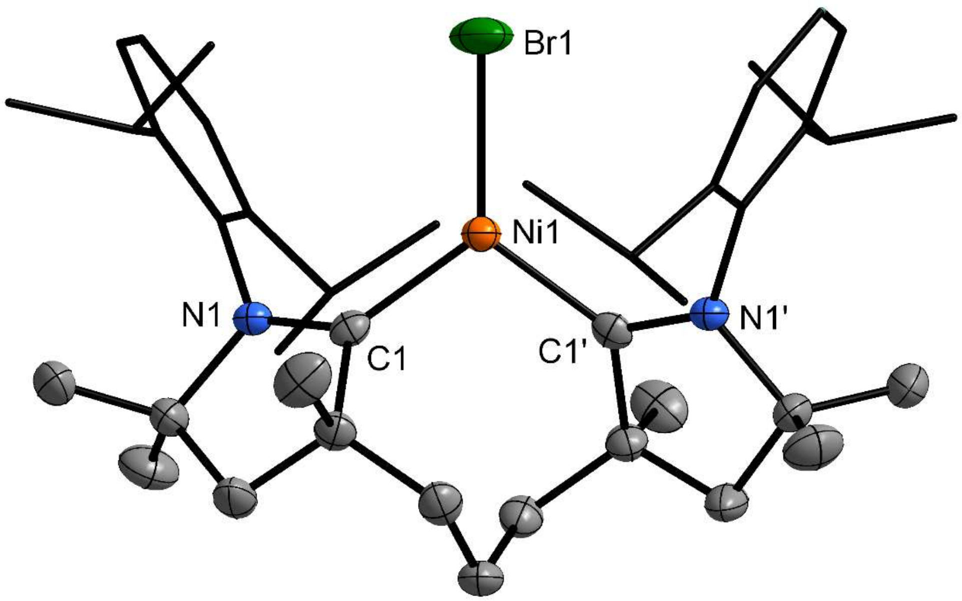

Supplementary Fig. 24. Solid-state structure of **9** with thermal ellipsoids drawn at 50 % probability and hydrogen atoms omitted for clarity. Selected bond lengths [Å] and angles [°]: Ni1-C1 1.922(3); Ni1-Br1 2.3381(8); C1-Ni1-C1’ 107.97(14).

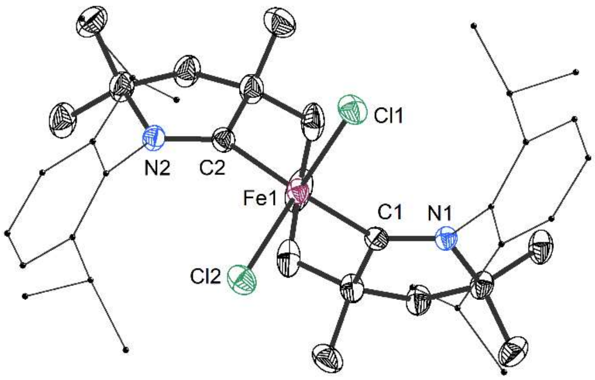

4

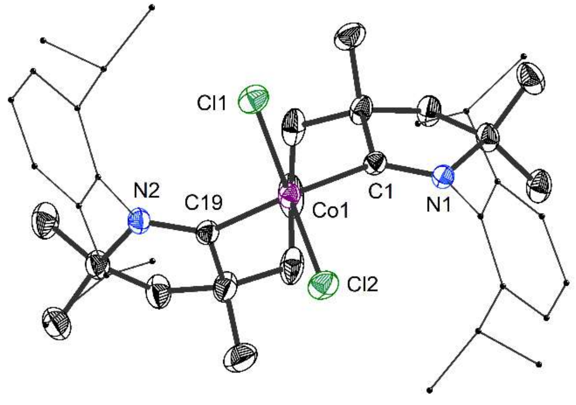

5

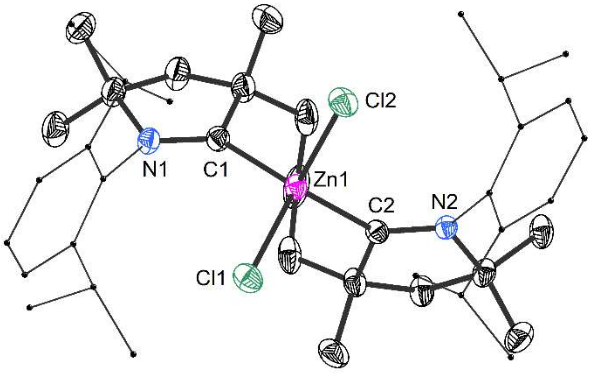

6

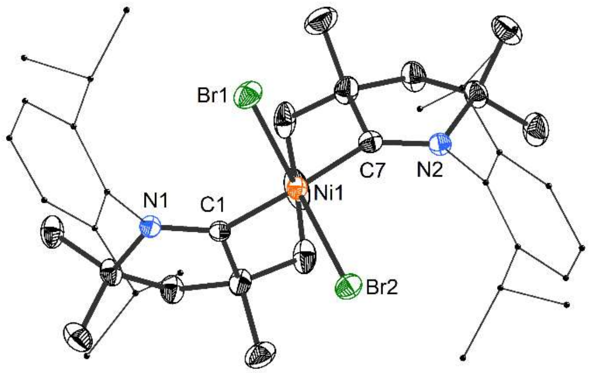

7

Supplementary Fig. 25. Solid-state structures of **4**–**7** viewed along the molecular (non-crystallographic) C_2-symmetry axis, with thermal ellipsoids drawn at 50 % probability and hydrogen atoms omitted for clarity.

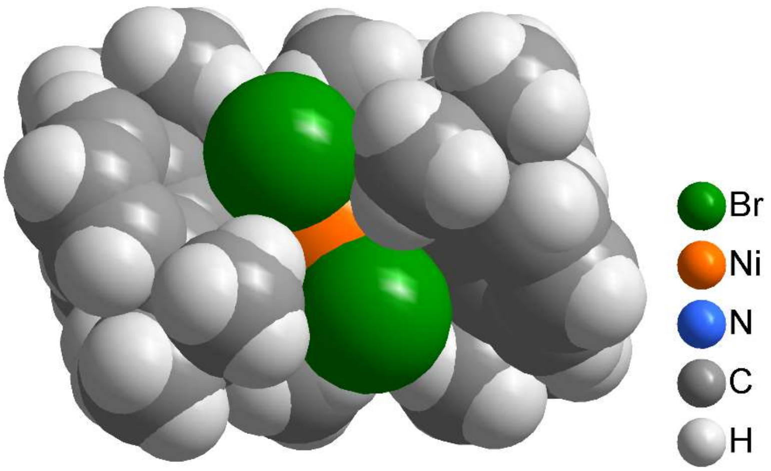

7

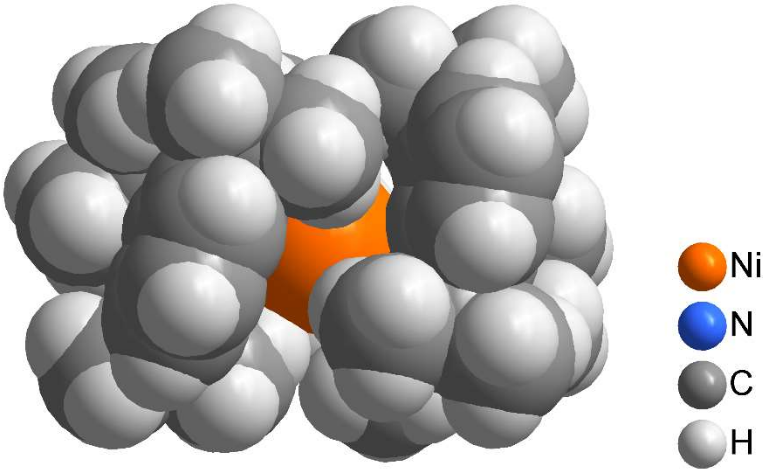

8

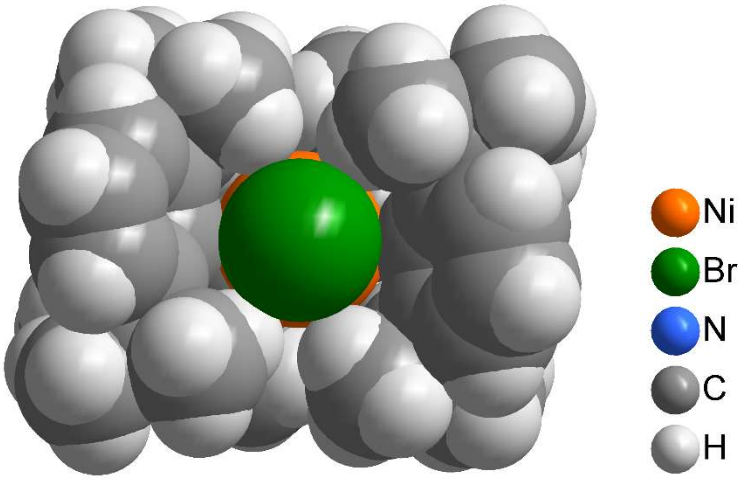

9

Supplementary Fig. 26. Space-filling representations of solid-state structures of **7** (top), **8** (middle), and **9** (bottom) viewed along the molecular (non-crystallographic) C_2-symmetry axis.

Computational Details

General Considerations

Geometries of bis(CAAC)Ni (**8**, both in C_1 and C_2 point group), $(CAAC)_2Ni$ (C_2), $(NHC)_2Ni$ (D_2), bis($CAAC^{Et}$)Ni (C_2), and bis($CAAC^{Bu}$)Ni (C_1), as well as those of the respective free ligands bis(CAAC) (C_2), CAAC (C_1), NHC (C_2), bis($CAAC^{Et}$) (C_2), and bis($CAAC^{Bu}$) (C_2) were optimized in the gas phase with dispersion corrected density functional theory. The well-performing theory-motivated PBE1PBE hybrid functional [7,8,9,10] was used together with sufficiently large but computationally feasible def2-TZVP basis sets [11] and Grimme's D3 dispersion correction with Becke-Johnson damping.[12,13] The structures were confirmed to be minima on the singlet potential energy hypersurface *via* calculation of the associated vibrational frequencies (all positive).

Metal–ligand bonding in the investigated nickel(0) complexes was analysed using the extended transition state (ETS) method for energy decomposition analysis (EDA).[14,15] These calculations employed the same PBE1PBE functional,[7,8,9,10] TZ2P STO basis sets,[16] Grimme's D3 dispersion correction with Becke-Johnson damping,[12,13] and treatment of scalar relativistic effects using the zero-order regular-approximated (ZORA) Hamiltonian.[17] The complexes were split into two fragments: the ligand(s) (bis(CAAC), 2 CAAC, 2 NHC, bis($CAAC^{Et}$), or bis($CAAC^{Bu}$)) and the standard spherical spin-restricted neutral nickel(0) atom in singlet state. While the latter is unphysical and does not correspond to the ground state of a nickel atom, it is a convenient choice for all complexes in question and allows the analysis of trends in bonding with respect to the identity of the ligand. No symmetry was used in these calculations.

All optimizations and frequency calculations were performed with the Gaussian 16-C.01 program suite,[18] while the ADF 2019.3 program package was used in analyses of metal–ligand bonding.[19] Unless stated otherwise, default settings for all key parameters, such as integration grids and convergence criteria, were used in all calculations.

Energy Decomposition Analyses

The EDA analysis (Supplementary Table 2) shows that the terms ΔE_{Pauli} (Pauli repulsion), ΔV_{elstat} (electrostatic interaction), ΔE_{orb} (orbital interaction), and ΔE_{disp} (dispersion) are largely independent of the complex in question. The obvious exception is $(NHC)_2Ni$, which has two poorly π-accepting ligands and, in contrast to crystal structure geometry, a linear C–Ni–C unit. When considering all CAAC-based complexes, the sum of the aforementioned four terms, ΔE_{int} (instantaneous interaction energy), becomes less negative as the C–Ni–C angle becomes more acute, but the variation is overall very small, save for the complex bis($CAAC^{Et}$)Ni with the smallest calculated bond angle. As shown in the literature,[20] the geometries of complexes L_2M (M = d^{10} element) can become non-linear owing to metal-to-ligand π-backdonation, which together with electrostatic interaction outweigh the general increase in Pauli repulsion upon bending. Connection to bond energy (ΔE_{bond}) is obtained by augmenting ΔE_{int} with the preparation energy (ΔE_{prep}) associated with changes in ligand geometry upon binding to the metal. As there is hardly any change in the structure of the CAAC ligand upon binding, ΔE_{bond} is the most negative for $CAAC_2Ni$, closely followed by bis($CAAC^{Bu}$)Ni and bis(CAAC)Ni in C_1 point group, that is, the two bis(CAAC) ligands having the least geometrical constrains.

Homologues of complex 8

A computational analysis of homologues of **8** incorporating 1,2-ethylene and 1,4-butylene linkers instead of the 1,3-propylene linker intrinsic to ligand **3** shows that their C–Ni–C bond angles are solely determined by geometric ligand constraints. The optimized structure of the complex having a short ethylene linker features a C–Ni–C bond angle of 127.1°, whereas the one having a longer butylene linker has a bond angle of 157.3°. The metal *d*-orbital manifold follows the expected trend (Supplementary Fig. 27) with d_{xz}- and d_{yz}-orbitals destabilizing and $d_{x^2-y^2}$- and d_{xy}-orbitals stabilizing upon narrowing of the angle at the metal (Supplementary Fig. 28), while the metal–ligand interaction energy remains virtually independent of the linker size (Supplementary Table 2). The ligand preparation energy is naturally the smallest for the butylene derivative, while that associated with the ethylene linker is on par with the value calculated for **8**.

Supplementary Table 2. Summary of Results from Energy Decomposition Analysis of Metal–Ligand Bonding in L_2Ni (L = NHC or CAAC) and LNi (L = bis(CAAC^{Bu}, bis(CAAC), or bis(CAAC^{Et})[a]

	L (point group)					
	NHC (D_2)	CAAC (C_2)	bis(CAAC^{Bu}) (C_1)	bis(CAAC) **8** (C_1)	bis(CAAC) **8** (C_2)	bis(CAAC^{Et}) (C_2)
ΔE_{Pauli}	1400	1547	1612	1633	1589	1616
ΔV_{elstat}	−1205	−1327	−1365	−1382	−1347	−1362
ΔE_{orb}	−743	−822	−838	−842	−833	−835
ΔE_{disp}	−44	−41	−43	−42	−40	−39
ΔE_{int}	−592	−644	−634	−633	−631	−620
ΔE_{prep}	27	10	16	33	45	30
ΔE_{bond}	−565	−634	−618	−600	−586	−590

[a] All energies are reported in kJ mol^{-1} and per two metal–ligand bonds.

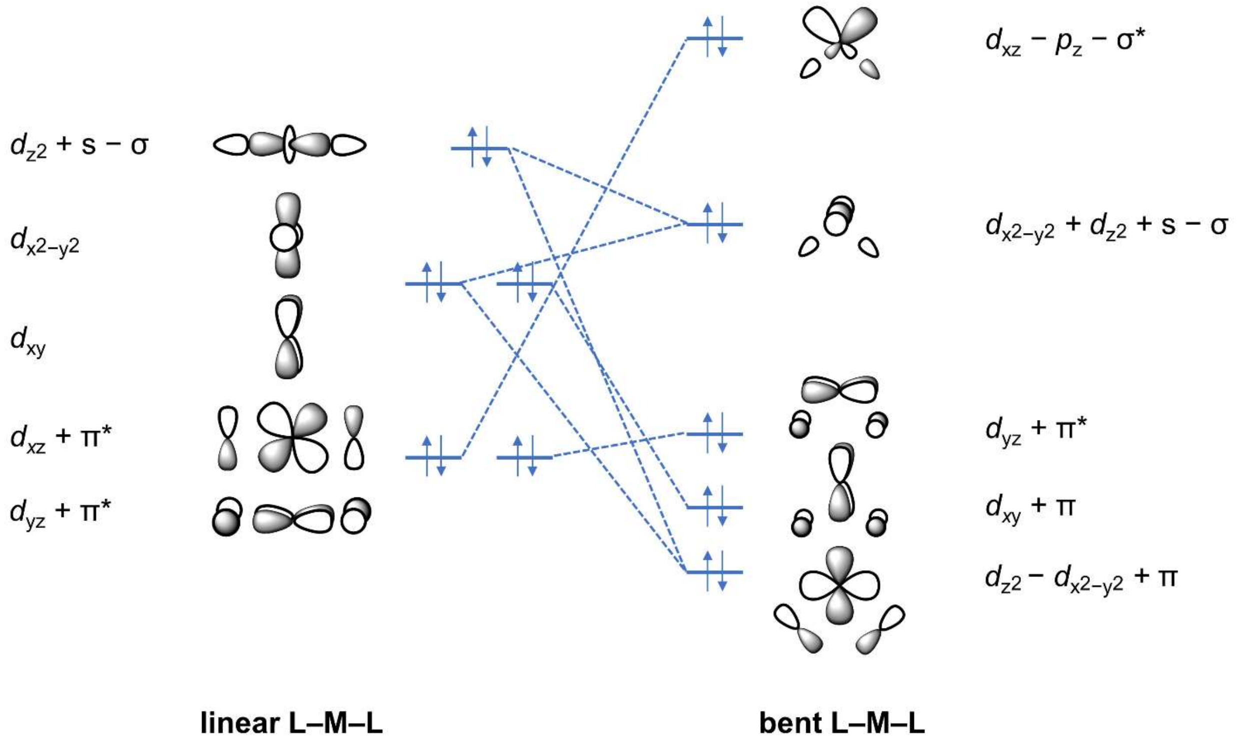

Supplementary Fig. 27. Simplified Walsh diagram for bending an L–M–L complex (L = π-acceptor ligand, M = d^{10}-metal). Adapted from Supplementary Reference 20.[20]

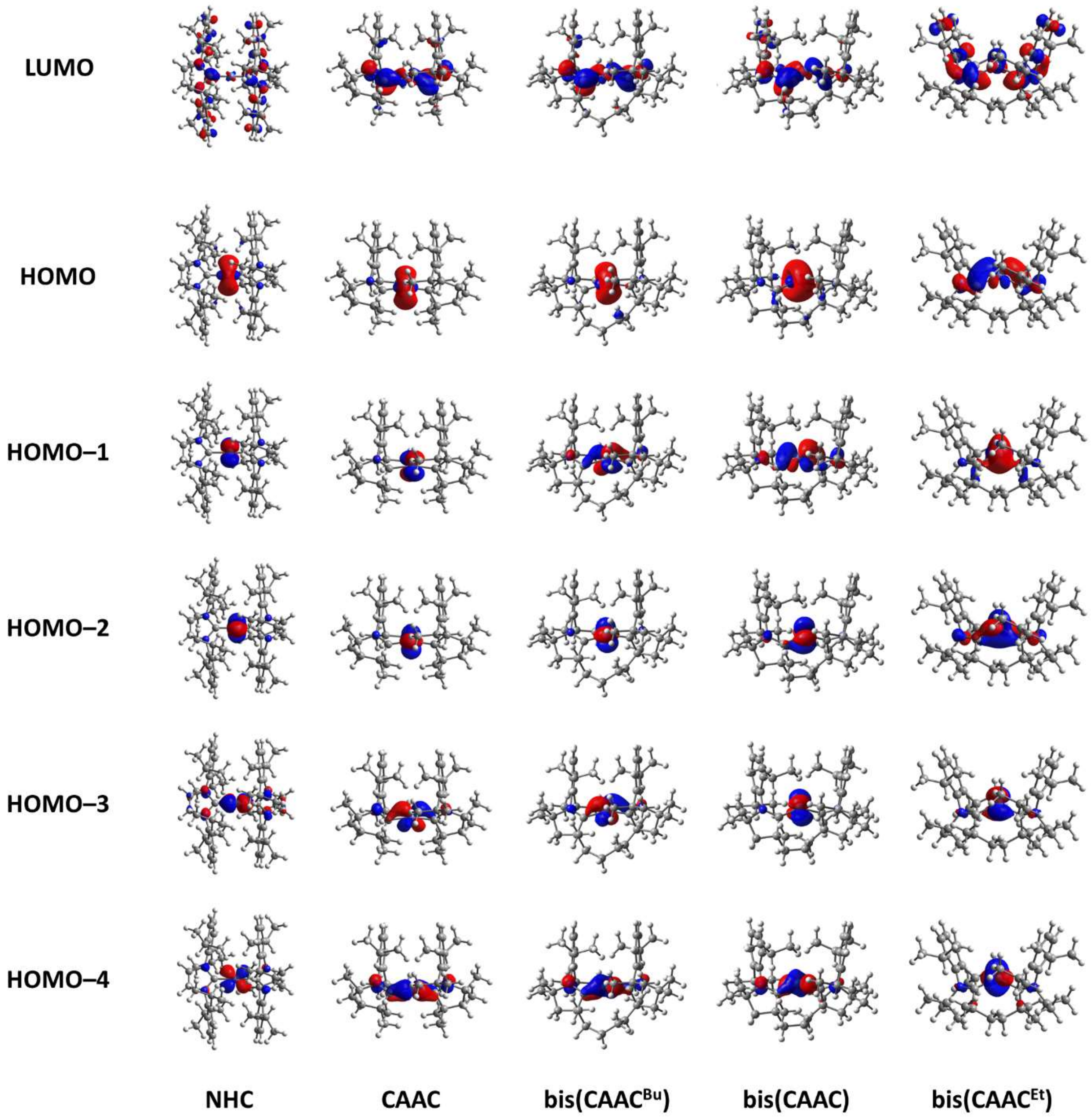

Supplementary Fig. 28. Canonical frontier Kohn-Sham orbitals of complexes L_2Ni (L = NHC or CAAC) and LNi (L = bis($CAAC^{Bu}$, bis(CAAC), or bis($CAAC^{Et}$).

Supplementary References

1. Chu, J., Munz, D., Jazzar, R., Melaimi, M. & Bertrand, G. Allene formation by gold catalyzed cross-coupling of masked carbenes and vinylidenes. *J. Am. Chem. Soc.* **138**, 7884–7887 (2016).
2. Dible, B. R., Sigman, M. S. & Arif, A. M. Oxygen-induced ligand dehydrogenation of a planar bis-μ-chloronickel(I) dimer featuring an NHC ligand. Inorg. Chem. *Inorg. Chem.* **44**, 3774–3776 (2005).
3. Fulmer, G. R. et al. NMR chemical shifts of trace impurities: Common laboratory solvents, organics, and gases in deuterated solvents relevant to the organometallic chemist. *Organometallics*, **29**, 2176–2179 (2010).
4. Sheldrick, G. M. SHELXT – Integrated space-group and crystal-structure determination. *Acta Cryst. A*, **71**, 3–8 (2015).
5. Sheldrick, G. M. Crystal structure refinement with SHELXL. *Acta Cryst. C*, **71**, 3–8 (2015).
6. Dolomanov, O. V., Bourhis, L. J., Gildea, R. J., Howard, J. A. K. & Puschmann, H. *J.* OLEX2: a complete structure solution, refinement and analysis program. *Appl. Cryst.* **42**, 339–341 (2009).
7. Perdew, J. P., Burke, K. & Ernzerhof, M. Generalized Gradient Approximation Made Simple *Phys. Rev. Lett.* **77**, 3865–3868 (1996).
8. Perdew, J. P., Burke, K. & Ernzerhof, M. Generalized Gradient Approximation Made Simple (Erratum) *Phys. Rev. Lett.* **78**, 1396 (1997).
9. Adamo, C. & Barone, V. Toward reliable density functional methods without adjustable parameters: The PBE0 model. *J. Chem. Phys.***110**, 6158–6170 (1999).
10. Ernzerhof, M. & Scuseria, G. E. Assessment of the Perdew–Burke–Ernzerhof exchange-correlation functional. *J. Chem. Phys.* **110**, 5029–5036 (1999).
11. Weigend, F. & Ahlrichs, R. Balanced basis sets of split valence, triple zeta valence and quadruple zeta valence quality for H to Rn: Design and assessment of accuracy. *Phys. Chem. Chem. Phys.* **7**, 3297–3305 (2005).
12. Grimme, S., Antony, J., Ehrlich, S. & Krieg, H. A. A consistent and accurate *ab initio* parametrization of density functional dispersion correction (DFT-D) for the 94 elements H-Pu. *J. Chem. Phys.* **132**, 154104/1–19 (2010).
13. Grimme, S., Ehrlich, S. & Goerigk, L. Effect of the damping function in dispersion corrected density functional theory. *J. Comput. Chem.* **32**, 1456–1465 (2011).
14. Kitaura, K. & Morokuma, K. A new energy decomposition scheme for molecular interactions within the Hartree-Fock approximation. *Int. J. Quantum Chem.* **10**, 325–340 (1976).
15. Ziegler, T. & Rauk, A. A theoretical study of the ethylene-metal bond in complexes between Cu^{+}, Ag^{+}, Au^{+}, Pt^{0}, or Pt^{2+} and ethylene, based on the Hartree-Fock-Slater transition-state method. *Inorg. Chem.* **18**, 1558–1565 (1979).
16. van Lenthe, E. & Baerends, E. J. Optimized Slater-type basis sets for the elements 1–118. *J. Comput. Chem.* **24**, 1142–1156 (2003).
17. van Lenthe, E., Baerends, E. J. & Snijders, J. G. Relativistic total energy using regular approximations. *J. Chem. Phys.* **101**, 9783–9792 (1994).
18. Frisch, M. J. et al. Gaussian 16, Revision C.01 (Gaussian, Inc., Wallingford CT, 2016).
19. ADF 2019.3, SCM, Theoretical Chemistry, Vrije Universiteit, Amsterdam, the Netherlands, http://www.scm.com.

20. Wolters, L. P. & Bickelhaupt, F. M. Nonlinear d^{10}-ML_2 transition-metal complexes. *ChemistryOpen* **2**, 106–114 (2013).
